# STAT3 or USF2 Contributes to HIF Target Gene Specificity

**DOI:** 10.1371/journal.pone.0072358

**Published:** 2013-08-21

**Authors:** Matthew R. Pawlus, Liyi Wang, Aya Murakami, Guanhai Dai, Cheng-Jun Hu

**Affiliations:** 1 Molecular Biology Graduate Program, School of Dental Medicine University of Colorado Anschutz Medical Campus, Aurora, Colorado, United States of America; 2 Department of Craniofacial Biology, School of Dental Medicine University of Colorado Anschutz Medical Campus, Aurora, Colorado, United States of America; 3 Institute of Basic Research, Zhejiang Academy of Traditional Chinese Medicine, Hangzhou, Zhejiang Province, China; University of Texas Southwestern Medical Center, United States of America

## Abstract

The HIF1- and HIF2-mediated transcriptional responses play critical roles in solid tumor progression. Despite significant similarities, including their binding to promoters of both HIF1 and HIF2 target genes, HIF1 and HIF2 proteins activate unique subsets of target genes under hypoxia. The mechanism for HIF target gene specificity has remained unclear. Using siRNA or inhibitor, we previously reported that STAT3 or USF2 is specifically required for activation of endogenous HIF1 or HIF2 target genes. In this study, using reporter gene assays and chromatin immuno-precipitation, we find that STAT3 or USF2 exhibits specific binding to the promoters of HIF1 or HIF2 target genes respectively even when over-expressed. Functionally, HIF1α interacts with STAT3 to activate HIF1 target gene promoters in a HIF1α HLH/PAS and N-TAD dependent manner while HIF2α interacts with USF2 to activate HIF2 target gene promoters in a HIF2α N-TAD dependent manner. Physically, HIF1α HLH and PAS domains are required for its interaction with STAT3 while both N- and C-TADs of HIF2α are involved in physical interaction with USF2. Importantly, addition of functional USF2 binding sites into a HIF1 target gene promoter increases the basal activity of the promoter as well as its response to HIF2+USF2 activation while replacing HIF binding site with HBS from a HIF2 target gene does not change the specificity of the reporter gene. Importantly, RNA Pol II on HIF1 or HIF2 target genes is primarily associated with HIF1α or HIF2α in a STAT3 or USF2 dependent manner. Thus, we demonstrate here for the first time that HIF target gene specificity is achieved by HIF transcription partners that are required for HIF target gene activation, exhibit specific binding to the promoters of HIF1 or HIF2 target genes and selectively interact with HIF1α or HIF2α protein.

## Introduction

Solid tumors are often hypoxic due to chaotic vascularization of the tumors and increased oxygen consumption of proliferating tumor cells and infiltrating inflammatory cells. Low oxygen concentration stabilizes the alpha subunits of hypoxia inducible factors (HIF1α or HIF2α). HIF1α and HIF2α translocate to nuclei where they associate with a constitutively expressed beta subunit called ARNT (Aryl Hydrocarbon Receptor Nuclear Translocator) or HIF1ß [1,2] to form HIF1α/ARNT (HIF1) or HIF2α/ARNT (HIF2) complexes. HIFs activate numerous hypoxia responsive genes by binding to HIF binding sites (HBS), a sub-region of hypoxia responsive elements (HREs) in the promoters or enhancers of HIF target genes. HIF target genes include genes that promote tumor cell growth, survival, invasion, angiogenesis and metabolism [3–6]. Thus, the hypoxic tumor microenvironment is a driving force for tumor progression [3–6].

The HIF1α and HIF2α subunits are considerably similar in the arrangement of their protein domains. The N-terminal half of HIF1α or HIF2α contains a basic DNA-binding domain and the ARNT interacting HLH/PAS domain while the C-terminal half of HIF1α or HIF2α contains an N-terminal transactivation domain (N-TAD), an inhibitory domain (IH), followed by a C-terminal transactivation domain (C-TAD) [7–11]. Most importantly, HIF1α and HIF2α exhibit significant similarities in several functional regions: they share 83%, 70% and 67% sequence identities in their basic DNA binding, HLH/PAS dimerization, and C-TAD domains.

Despite the similarities between HIF1α and HIF2α in protein domain structure, primary protein sequence, dimerization partner and regulation of protein stability by oxygen concentration, HIF1 and HIF2 activate transcription of different sets of hypoxia responsive genes both in cultured cells or in animals where both HIF1α and HIF2α are expressed [12–21]. Interestingly, the target gene specificity observed between HIF1 and HIF2 transcription factors is not determined by specific binding of HIF1 (or HIF2) to HIF1 (or HIF2) target genes as several reports have demonstrated detection of similar amount of both HIF1α and HIF2α protein on most promoters of HIF target genes [12,22–24]. The lack of binding specificity of HIF1 and HIF2 to the promoters of HIF target genes is also confirmed by functional studies using over-expressed HIF. For example, constitutively active HIF1α can activate cloned and endogenous HIF2 specific target genes *PAI1* and *EPO* [12,13,22]. Additionally, constitutively active HIF2α can also activate cloned and endogenous HIF1 specific target gene promoters of *PGK1* and *CA9*, albeit weakly [12,22]. Furthermore, both HIF1 and HIF2 can activate artificial reporters with multiple copies of HBS from a HIF1 target gene *PGK1* [12,13]. Additional studies have demonstrated that HIF target gene specificity is determined by post-DNA binding mechanisms since switching of the basic DNA binding domain of HIFα does not change the target gene specificity [12,22]. More specifically, these reports also indicate that the HIFα N-TAD contributes to HIFα target gene specificity, possibly via specific interaction with co-activators or other transcription factors [12,22].

Activation of hypoxia responsive genes, like transcription of other eukaryotic genes requires multiple transcription factors which recruit chromatin remodeling complexes, histone modifying enzymes and basal transcriptional machinery to form stable “enhanceosome” complexes [25]. The importance of HIF1 and HIF2 in activating hypoxia responsive genes is well established as inhibiting HIF activity prevents hypoxic induction of HIF target genes [12,13,19,26,27]. However, the identities of other transcription factors required for global hypoxic activation of HIF target genes are less clear. We have recently identified two transcription factors, STAT3 (Signal Transducer and Activator of Transcription 3) and USF2 (Upstream Stimulatory Factor 2) that are required for HIF target gene induction during hypoxia [28,29]. In these previous studies, using siRNA knockdown or specific inhibitor we showed that STAT3 is specifically required for hypoxic induction of HIF1 target genes in MDA-MB-231 and RCC4 cells [28] while USF2 is required for hypoxic induction of HIF2 target genes in Hep3B, RCC4T and PRC3 cells [29]. STAT3 or USF2 activate HIF1 or HIF2 target genes partly by binding to HIF1 or HIF2 target gene promoters and recruiting histone acetylases p300 and CBP [28,29]. In this report, we probed the role of STAT3 and USF2 in HIF target gene specificity. Our findings that HIF transcription partners contribute to HIFα target gene specificity have important implications for our understanding of HIF1 and HIF2-mediated gene expression in response to hypoxia, which is important in cancer biology

## Materials and Methods

### Cell culture

HEK293T, RCC4 and RCC4T (ATCC) cells were grown in high-glucose DMEM (Hyclone) with 10% FBS. Hep3B cells (ATCC) were cultured in MEM/EBSS (Hyclone) containing 10% FBS. For hypoxic treatment, 25 mM HEPES was added to growth media and cells were incubated at 21% or 1.2% O_2_ for 16 hr (or otherwise noted). RCC4 or Hep3B cells stably knockdown of STAT3 or USF2 were described previously [28,29].

### Plasmid construction

PGK1/Luc and PAI1/Luc reporters have been described [12,29]. The CA9 (-1096/+25) promoter, EPO promoter (-976/+56) and enhancer (+2511/+3111) were amplified from human genomic DNA using GC-Melt genomic DNA polymerase (Clontech) and inserted into the pGL3basic luciferase vector (Promega). The CA9 2HBS/Luc, CA9 2USF2V1/Luc and CA9 2USF2V2/Luc constructs were made by PCR-mediated protocol that added two -191 HBSs from human PAI1 promoter, or added -638 and -546 USF2 sites from PAI1 promoter near -13 HBS or -1096 location of CA9/Luc (-1096/+25). The reporters of CA9 -506/+25 and CA9 -171/+25 were made by PCR-mediated deletion of -1096 to -506 or -1096 to -171 regions from the CA9 -1096/+25/Luc reporter. The CA9 -506/+25 HBS/HBS/Luc was made by replacing the original -13 HBS in CA9 promoter with the -191 HBS from PAI1 promoter while CA9 -506/+25 STAT3/HBS/Luc and CA9 -506/+25 STAT3/USF2/Luc were generated by PCR-mediated replacement of STAT3 binding sites in the CA9 promoter with -191 HBS or USF2 binding sites from the PAI1 promoter. The CA9 -506/+25HBS/HBS+STAT3/USF2/Luc was made by PCR-mediated replacement of STAT3 binding sites in the CA9 promoter with USF2 binding sites from PAI1 promoters using construct 4 DNA as template in PCR. All the constructs were sequenced to verify the intended changes. The pcDNA3.1hHIF1αTM-Flag (triple mutations of P402A/P577A/N813A of human HIF1α protein with Flag-tag) and pcDNA3.1hHIF2αTM-Flag have been described previously [12] and their deletion mutants were generated by PCR-based mutagenesis using the full-length hHIF1αTM-Flag or hHIF2αTM-Flag as templates. The pcDNA3.1 STAT3C-HA (constitutive active STAT3 with HA-tag at C-terminus) was described previously [28]. The USF2, mHIF1αDPA (double proline to alanine) and mHIF2αDPA, and HIF1α/HIF2αDPA hybrid expression plasmids were described previously [12,15].

### Transient transfection

A. Reporter assay. All promoter reporter assays were conducted using Fugene 6 Transfection Reagent (Roche) to transfect DNA into HEK293T cells. Typically, ~50% confluent HEK293T cells in one well of the 6-well plates were transfected with 200 ng reporter DNA and 200 ng β-galactosidase. In addition, 200 ng HIFα (or HIF1α/HIF2α hybrid plasmid), USF2, or STAT3C expression vector, or 200 ng each of HIFα+USF or STAT3C were co-transfected to activate the reporter genes. 36 hours after transfection, cells were collected into 400 µl 1x Reporter Lysis Buffer (Promega) and assayed for β-gal activity and luciferase activity using a luminometer. Promoter activation by HIFα, USF, or STAT3 or combination of HIFα/USF2 or STAT3 was corrected for β-gal transfection efficiency and presented as fold of induction relative to promoter activities from an empty control vector. Results were the average of at least three experiments. B. Transfection of Hep3B to assess endogenous target gene activation. 60% confluent Hep3B cells in 6 cm dishes were transfected with 3 µg of HIFαTM or USF2, or STAT3C DNA or 1.5 µg each of HIFαTM+USF2 (or STAT3C) using Lipofectamine and Lipofectamine Plus Reagent (Invitrogen). RNA was collected from cells 48 hrs post-transfection to analyze the HIF target gene expression. Results were the average of at least three experiments.

### RNA preparation and reverse transcription-quantitative PCR (RT-qPCR) to analyze mRNA levels and q-PCR for ChIP

RNA was isolated from cells using the Qiagen RNeasy-Plus kit utilizing DNase to digest possible contaminated genomic DNA. RNA was reverse-transcribed using the ISCRIPT Advanced Reverse Transcription Kit (BioRad). Levels of mRNA were quantified by SYBR Green qPCR using the CFX384 Real-Time System (BioRad). All primer sets designed to detect target gene mRNA or used in ChIP were validated for their product specificity and amplification efficiency using melt curve analysis, qPCR product sequencing, and standard dilution analysis. Amplification efficiencies of primer sets were between 90 and 110%. qPCR results for mRNA were normalized using both 18S rRNA and beta-actin mRNA. qPCR for ChIP DNA was performed using primers located near validated HRE positions of genes or in intron4 of the PAI1 gene as a negative control. Results were the average of a minimum of three independent experiments performed in triplicate. Primer sequences for mRNA and DNA ChIP can be found in Table S1.

### Co-Immunoprecipitation

For STAT3 and USF2 interaction with HIFα protein in over-expression setting, HEK293T cells were transfected with HA-tagged constitutively active full-length (FL) STAT3C or HA-tagged wild type USF2 alone, or STAT3C-HA (or USF2-HA) with Flagged constitutively active full-length or deletion mutants of HIF1αTM or HIF2αTM. Lysates from transfected 293T cells were immunoprecipitated with anti-Flag M2 beads (Sigma) to pull-down Flag-tagged HIF1α or HIF2α protein, in which HA-tagged STAT3 or USF2 was detected. For endogenous STAT3 and USF2 interaction with HIFα, STAT3 (or USF2) protein was precipitated by anti-STAT3 (or anti-USF2) antibodies and protein A/protein G beads. The co-precipitated HIF1α or HIF2α protein was detected using anti-HIFα antibodies.

### ChIP and ChIP/ReChIP

ChIP assays were performed as described [29]. The genomic DNA was sonicated to an average of 500bp, thus qPCR primer were able to detect a much larger region than their designated amplicon. Anti-HIF1α (NB 100-134B3, Novus), anti-HIF2α (NB 100-122, Novus), anti-USF2 (C-20, SC-862, Santa Cruz), anti-STAT3 (K-15, sc-483; Santa Cruz), and anti-POL II (H-224, SC-9001, Santa Cruz) antibodies were used for protein-DNA complex precipitation, whereas rabbit preimmune serum served as a background control. DNA from input or immunoprecipitated genomic DNA was assayed using SYBR-Green based qPCR (BioRad, Real-Time Detection System) with primers designed to amplify the *CA9*, *PGK1*, *PAI1*, *EPO* or *VEGF* promoter around the reported HREs or in intron 4 of PAI1 for negative control. ChIP/ReChIP: ChIP was performed as above, binding complexes from the first IP were eluted from the sepharose beads using re-ChIP buffer. The eluted protein/DNA complexes were diluted in RIPA buffer and re-subjected to ChIP using a different antibody [29].

### Protein Analysis

Typically whole cell lysates were used for western blot analysis. Protein concentration was determined by BCA protein assay kit (Pierce, Prod# 23223 and 23224) and the same amount of protein was loaded in western blot. Western blot analysis was performed using standard protocols with the following primary antibodies: anti-HIF1α (NB 100-105, Novus Biologicals), anti-HIF2α (NB100-122, Novus Biological), anti-STAT3 (total) (K-15, sc-483, Santa Cruz), anti-USF2 (C-20, SC-862, Santa Cruz), anti-Flag (F-3165, Sigma), anti-HA (MMS-101P, Covance) and anti-beta actin (Sc-1616, Santa Cruz).

### Statistical analysis

Two-tail t-test were performed for this paper with “*” indicating p<0.05 and “**” indicating p<0.01. All the controls for the t-tests were indicated in the figures.

## Results

### Over-expressed USF2 specifically activates cloned and endogenous HIF2 target genes

**Figure 1:**
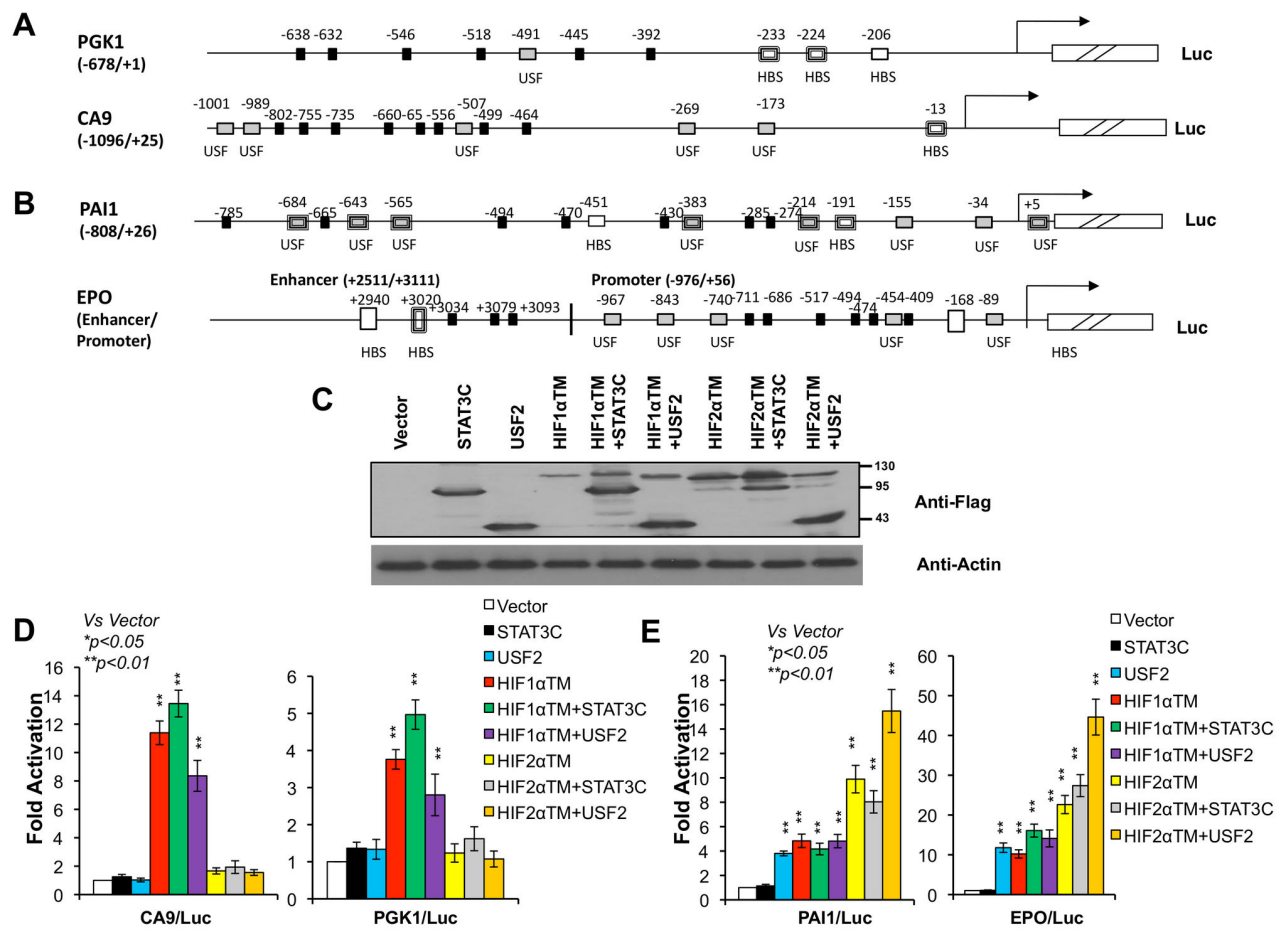
STAT3 or USF2 alone or with HIF1α or HIF2α to activate the cloned promoters of HIF1 or HIF2 target genes in 293T cells. **A**) Schematic presentation of the promoters of HIF1 target genes *PGK1* and *CA9*. **B**) Schematic presentation of the promoters/enhancers of HIF2 target genes *PAI1* and *EPO*. Predicted STAT3 {TT(N) _4-6_AA} binding sites (black solid boxes), USF binding sites (CANNTG) (gray boxes), and HIF binding sites (HBS, ACGTG) (white boxes) are indicated. Previously validated HIF and USF2 binding sites are indicated by bold boxes. **C**) Western blot analysis of Flag-tagged STAT3C, USF2, HIF1αTM and HIF2αTM to monitor the expression of these plasmids in reporter gene assays for Figure 1D-E. Anti-beta actin was for loading control of total protein for this figure and others in the study. **D**) Fold of induction of CA9/Luc and PGK1/Luc reporters activated by the indicated plasmids. **E**) Fold of induction of PAI1/Luc and EPO/Luc reporters activated by the indicated plasmids.

Using siRNA or inhibitor to reduce endogenous STAT3 or USF2 activity, we previously showed that STAT3 or USF2 is specifically required to promote HIF1 or HIF2 target gene expression during hypoxia by binding to the promoters of HIF1 or HIF2 target genes and recruiting histone acetylases p300 and CBP to promoters of HIF1 or HIF2 target genes [28,29]. However, these previous experiments did not test if STAT3 can bind to HIF2 target gene promoters or if USF2 can bind to HIF1 target gene promoters. Additionally, our previous experiments did not test if over-expressed STAT3 or USF2 results in a loss of target gene specificity, as is the case with over-expression of HIF1α and HIF2α. We first analyzed the promoter and enhancer regions of two HIF1 target genes (*CA9* and *PGK1*) and two HIF2 target genes (*PAI1* and *EPO*) for potential binding sites of STAT3 {TT(N) _4-6_AA} [30] and of USF2 (CANNTG) [31] (Fig. 1A-B). Unexpectedly, we found similar numbers of potential STAT3 binding sites on the promoters of HIF1 and HIF2 target genes with an average frequency of 8 potential sites per 1 Kb of promoter DNA (Fig. 1A-B). Again, similar numbers of potential USF2 binding sites were found on the *CA9* promoter, a HIF1 target gene, and the *EPO* promoter, a HIF2 target gene. However, the *PAI1* promoter, a HIF2 target gene, contained more USF2 binding sites than the promoter of the HIF1 target gene *PGK1* (Fig. 1A). To test the binding specificity of STAT3 and USF2 on the promoters of HIF1 and HIF2 target genes, we first used a functional assay to test if over-expressed STAT3 or USF2 activated promoters of HIF target genes distinctly. We constructed CA9/Luciferase (Fig. 1A) and EPO/Luciferase (Fig. 1B) reporter genes since their functional HIF binding sites (HBS) were previously determined at -13 and +3020 from the transcription start site respectively (Fig. 1A-B) [32,33]. These newly-generated reporters and our previously described reporters of PGK1/Luc [12] and PAI1/Luc [29] were co-transfected with 200 ng DNA expressing no protein (Vector), constitutively active STAT3 (STAT3C), or USF2 into 293T cells. Although STAT3 activity increases HIF1 target gene induction in RCC4 and MDA-MB-231 cells by at least 2 fold [28], we were surprised to find that the HIF1 target gene promoters of the *CA9* and *PGK1* were not or were only weakly activated by STAT3C (Fig. 1D, the increase was not statistically significant for PGK/Luc) while HIF2 target gene reporters were not activated by STAT3C at all (Fig. 1E). Weak activation (1.5 fold) of a *VEGF* reporter, a known STAT3 target gene, was also observed (data not shown), indicating STAT3 was not able to strongly activate its target gene promoters alone under this setting. 293T cells exhibited active endogenous STAT3 (data not shown), co-transfected STAT3C also only weakly activated these STAT3 target promoters when 293T cells were treated with the STAT3 inhibitor S3I-201 which was expected to inhibit endogenous, but not STAT3C activity (data not shown). These results indicated that endogenous active STAT3 activity is not responsible for the weak activity of transfected STAT3C in regulating these reporters in 293T cells. Importantly, USF2 strongly activated HIF2 target gene reporters (Fig. 1E), but not HIF1 target gene reporters (Fig. 1D), indicating USF2 exhibited HIF2 target gene specificity even when it was over-expressed. Of note, STAT3C and USF2 protein were similarly expressed as both plasmids were Flag-tagged (Fig. 1C), indicating that weak activation of HIF1 target genes by STAT3C was not due to its low expression.

**Figure 2:**
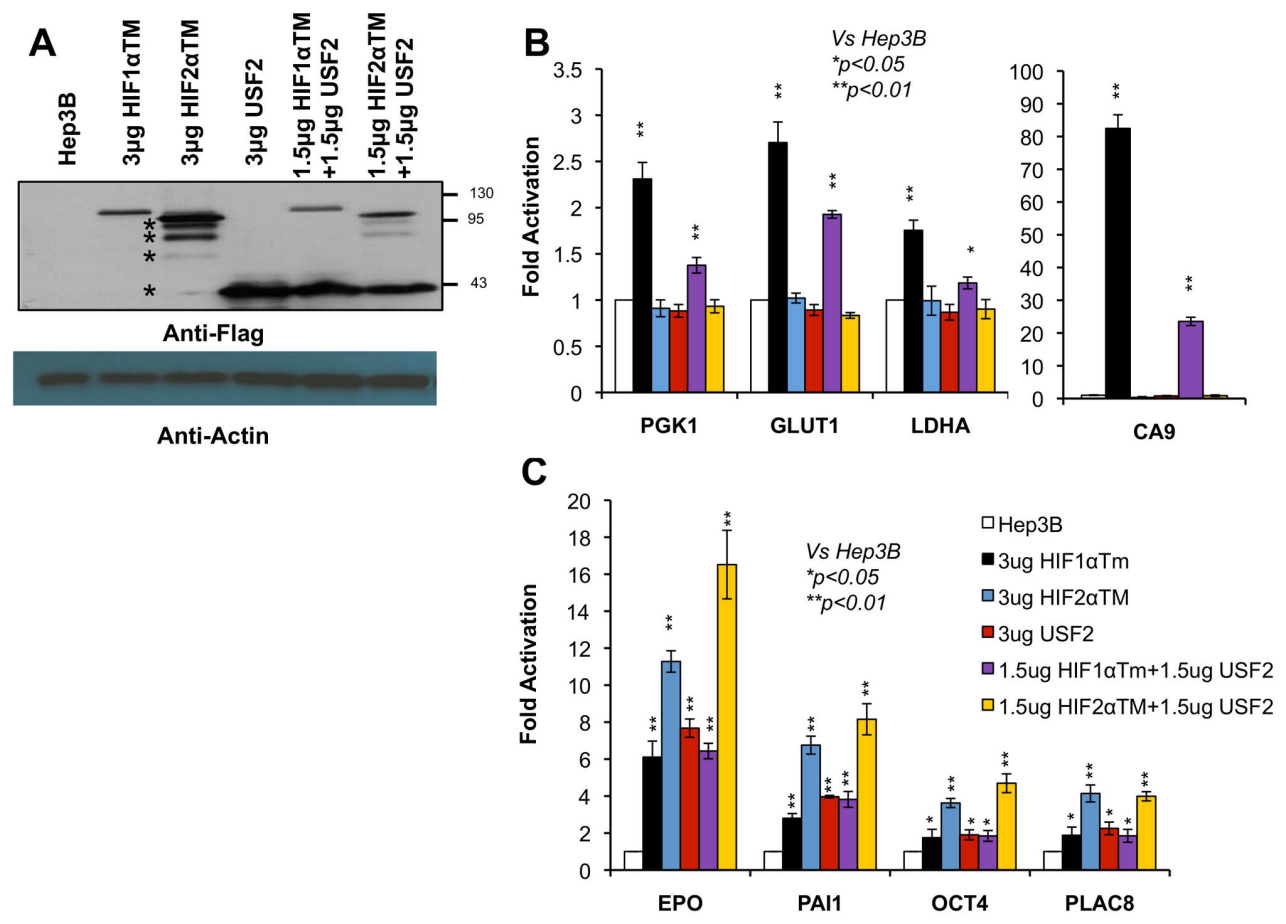
USF2 functions alone or with HIF2α to activate endogenous HIF2 target genes in normoxic Hep3B cells. **A**) Western blot analysis of Flag-tagged USF2, HIF1αTM and HIF2αTM to monitor the expression of these plasmids in transfected Hep3B cells. The starts * indicate several HIF2α protein bands expressed from the vector. **B**) mRNA levels of HIF1 target genes, *PGK1*, *GLUT1*, *LDHA* and *CA9*, in normoxic Hep3B cells in response to transient transfection of the indicated plasmids. **C**) mRNA levels of HIF2 target genes, *EPO*, *PAI1*, *OCT4* and *PLAC8*, in normoxic Hep3B cells in response to transient transfection of the indicated plasmids.

Consistent with the reporter gene assays, STAT3C was not able to activate endogenous HIF1 or HIF2 target genes or known STAT3 target genes such as *VEGF* and *MYC* in Hep3B cells (data not shown) although there is a relatively low amount of active endogenous STAT3 in Hep3B cells [29]. Interestingly, over-expression of USF2 activated endogenous HIF2 target genes including *EPO*, *PAI1*, *OCT4* and *PLAC8* (Fig. 2C), but not HIF1 target genes of *PGK1*, *GLUT1*, *LDHA* and *CA9* in Hep3B cells (Fig. 2B). To directly test if DNA binding was required for USF2 to activate cloned or endogenous HIF2 target genes, similar experiments were performed using the USF2∆basic construct in which the basic DNA binding domain of USF2 was deleted. USF2∆basic was unable to activate cloned or endogenous HIF2 target genes (data not shown). In summary, these data for the first time demonstrated that over-expressed USF2 exhibited specific binding to HIF2, not HIF1 target genes by activation assays.

### STAT3 preferentially binds to promoters of HIF1 target genes while USF2 preferentially binds to promoters of HIF2 target genes

**Figure 3:**
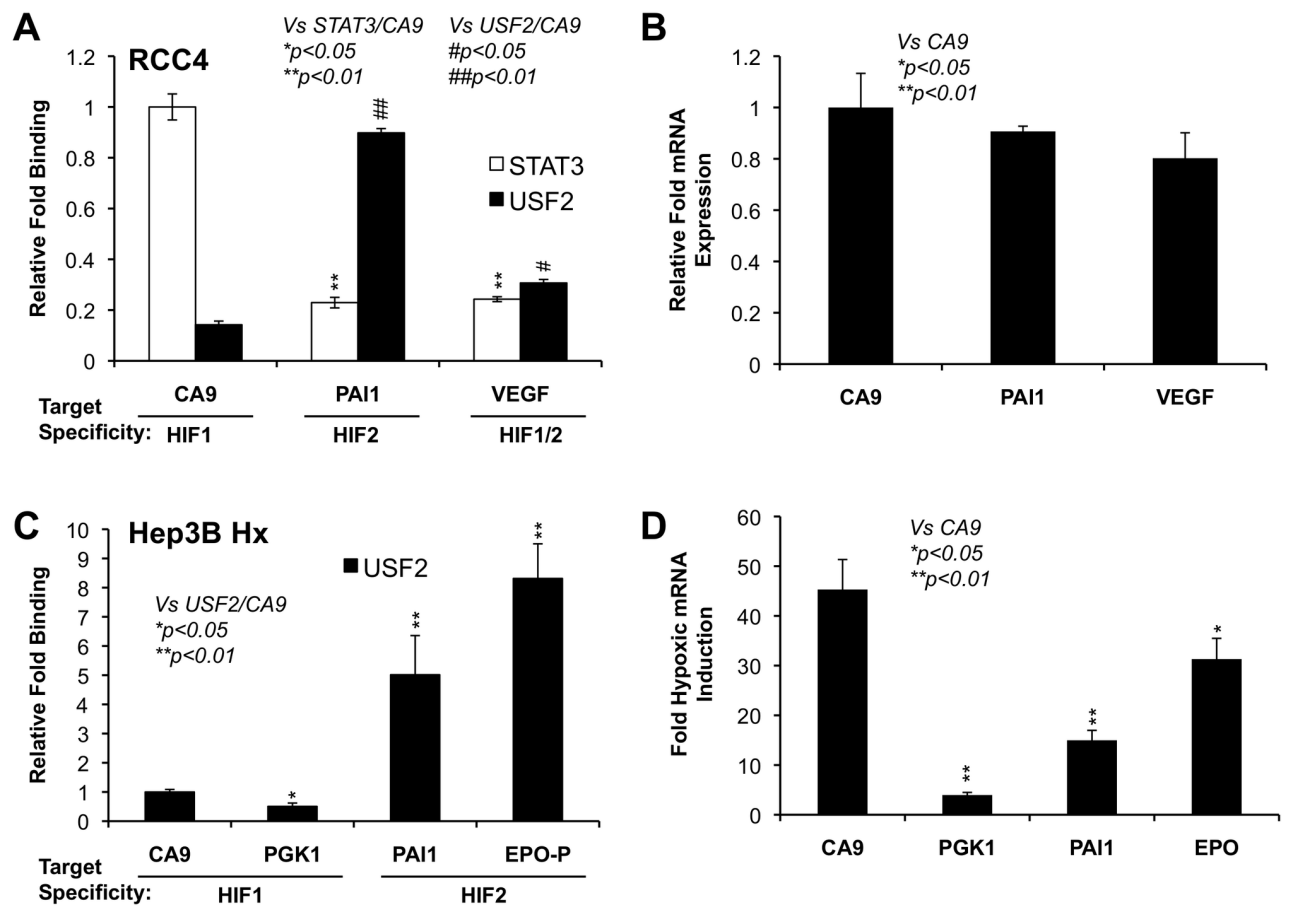
STAT3 and USF2 are enriched on the promoters of HIF1 or HIF2 targets respectively. Chromatin immunoprecipitation was performed in chromatin lysates from normoxic RCC4 (**A**) or hypoxic Hep3B cells (**C**). Antibodies against STAT3 or USF2 were used to co-precipitate STAT3- and USF2-associated genomic DNA. Q-PCR was used to detect the promoters of HIF1 target genes, *CA9* and *PGK1*, the enhancer or promoter of HIF2 target genes, *EPO* and *PAI1*, or the HIF1/HIF2 common target, *VEGF*. Results were normalized to input samples as % of input. The relative fold binding was calculated by dividing each of the % input values by the % input value of STAT3 associated with CA9 promoter here in Figure 3A. Similar calculations were made for Figure 3C and ChIP/Re-ChIP in Figure 9. **B**) The relative expression levels of *CA9*, *PAI1* and *VEGF* mRNAs in normoxic RCC4 cells. **D**) The fold of induction of HIF1 target genes *CA9* and *PGK1*, HIF2 target genes *PAI1* and *EPO* in hypoxic Hep3B cells.

We were unable to check STAT3’s binding specificity using gene activation (Figs. 1 and 2). Thus, we tested STAT3 binding specificity using chromatin immuno-precipitation (ChIP). At the same time, ChIP was also performed for USF2 to confirm USF2’s binding specificity as suggested by reporter assays (Figs. 1 and 2). STAT3 and USF2 ChIP experiments were conducted in normoxic pVHL-deficient RCC4 cells, a cell line in which STAT3 or USF2 is required specifically for HIF1 or HIF2 target gene activation respectively [28,29]. While STAT3 and USF2 were able to bind to the promoters of both HIF1 and HIF2 target genes (Fig. 3A), STAT3 bound more abundantly on the promoter of a HIF1 target gene, *CA9* (Fig. 3A) than on the promoter of a HIF2 target gene, *PAI1* (Fig. 3A) in RCC4 cells. In contrast, USF2 bound more abundantly on the promoter of a HIF2 target, *PAI1* than on the promoter of a HIF1 target gene, *CA9* (Fig. 3A). Importantly, both STAT3 and USF2 exhibited similar binding profiles on the promoter of a HIF1/HIF2 common target gene, *VEGF* (Fig. 3A). The different levels of STAT3 or USF2 binding on the promoters of *CA9*, *PAI1* and *VEGF* was not due to different levels of gene expression as these genes were similarly expressed in RCC4 cells, which was determined by cycle threshold (Ct) from qPCR (Fig. 3B). Importantly, in these and subsequent ChIP experiments, only a negligible amount of protein binding was observed to negative control region at intron 4 of the PAI1 gene (data not shown), demonstrating specific protein binding to the indicated genomic regions. We also detected low/negligible genomic DNA precipitated by pre-immuno serum (data not shown).

Hypoxic activation of HIF2, but not HIF1 target genes in Hep3B cells is USF2 dependent [29]. To further confirm the binding specificity of USF2, USF2 ChIP experiments were also conducted in hypoxic Hep3B cells. Interestingly, USF2 binding on the promoters of the HIF2 target genes *PAI1* and *EPO* was much greater than USF2 binding on the promoters of the HIF1 target genes, *CA9* and *PGK1* (Fig. 3C). Again, the reduced levels of USF2 binding on the promoters of the HIF1 target gene *CA9*, was not due to lack of activation since *CA9* was strongly induced by hypoxia (Fig. 3D). However, we were not able to test the STAT3 binding specificity in Hep3B cells since STAT3 is not activated in Hep3B cells by hypoxia, nor involved in HIF1 target gene induction in Hep3B cells, indicating that unknown transcription factors are involved in HIF1 target gene activation in Hep3B cells [28]. These data confirmed our previous findings that STAT3 and USF2 bind to the promoters of HIF1 or HIF2 target genes, and also for the first time provided direct evidence that that STAT3 or USF2 does not bind to the promoters of HIF2 or HIF1 target genes, respectively, in RCC4 or Hep3B cells despite the fact that these HIF1 or HIF2 target genes are expressed and have similar numbers of potential STAT3 and USF2 binding sites.

### There is a specific functional interaction of STAT3 with HIF1α on HIF1 target genes and USF2 with HIF2α on HIF2 target genes in an over-expression system

We previously established a specific functional interaction between USF2 and HIF2α on HIF2 [29] and STAT3 and HIF1α on HIF1 target genes [28] using knock-down of endogenous STAT3 or USF2 activity and using endogenous HIF target gene mRNA as a readout. Over-expression of HIF led to loss of HIF target gene specificity [12,13,22] (also see below). The maintenance of specific binding of over-expressed STAT3 or USF2 on the promoters of HIF1 or HIF2 target genes establishes a foundation for us to test here if the functional interaction specificities were maintained in a system in which both STAT3/USF2 and HIF1α/HIF2α are over-expressed. Thus, the two HIF1 target gene reporters, CA9/Luc and PGK1/Luc, as well as the two HIF2 target gene reporters, PAI1/Luc and EPO/Luc, were transfected into HEK293T cells with 200 ng of HIF1αTM (triple mutation to make HIF1α stable and active under normoxia), HIF2αTM, STAT3C, or USF2 or 200ng each of two activators: HIF1αTM+STAT3C, HIF1αTM+USF2, HIF2αTM+STAT3C, or HIF2αTM+USF2. Consistent with previous results [12,22], the HIF1 target gene promoters of *CA9* and *PGK1* were activated strongly by over-expressed HIF1αTM and also by HIF2αTM albeit weakly (around 1.5 fold, Fig. 1D) while the HIF2 target gene promoter/enhancers, *PAI1* and *EPO*, were activated by both HIF2α and HIF1α (Fig. 1E) although HIF2 activated these HIF2 target genes better than HIF1α (Fig. 1E). Interestingly, activation of HIF1 target gene reporters by HIF1α was enhanced by co-transfection of STAT3C (Fig. 1D, P < 0.05 vs HIF1α only), but reduced by co-transfected USF2 (Fig. 1D, P < 0.05 vs HIF1α only). This inhibitory role of USF2 for some HIF1 target genes is consistent with previous reports indicating that HBSs of some HIF1 target genes, such as *LDHA*, *BNIP3*, and *LPK*, are bound by USF1/USF2 under normoxia but by HIF1 in hypoxic cells [34] as we also observed previously [29]. The weak activation of HIF1 target gene reporters by HIF2 was not changed by co-transfection of STAT3 or USF2 (Fig. 1D). For HIF2 target gene reporters, their activation by HIF1 was not changed by co-transfection of STAT3 or USF2 (Fig. 1E). However, activation of the HIF2 target gene reporters by HIF2α was enhanced by co-transfection of USF2 (Figure 1E, P < 0.05 vs HIF2α only), but not changed by STAT3C (Figure 1E). Importantly, we were able to monitor the protein levels of these four plasmids in relation to one another since they were flag tagged (Figure 1C).

Consistent with the reporter assay, endogenous HIF1 target genes *PGK1*, *GLUT1*, *LDHA* and *CA9* were mainly activated by HIF1α, not by HIF2α (Fig. 2B) while HIF2 target genes of *EPO*, *PAI1*, *OCT4* and *PLAC8* were activated by both HIF1α and HIF2α (Fig.2C). Consistent with reporter genes (Fig.1D), co-transfection of USF2 reduced the activation of these HIF1 target genes by HIF1α (Fig. 2B). Co-transfection of USF2 increased the ability of HIF2α to activate the HIF2 target gene, *EPO*, but not *PAI1*, *OCT4* and *PLAC8* (Fig. 2C) despite our previous report indicated that hypoxic induction of all these genes was significantly reduced in Hep3B/USF2 knockdown cells [29] (see discussion). Although cells transfected with HIF1α+USF2 expressed less HIF1α protein than cells transfected with HIF1α only, the reduced activation of HIF1 target genes by HIF1α+USF2 (Fig. 2B) was likely due to co-transfected USF2 since HIF1α+USF2 did not reduce the activation of HIF2 target genes compared to cells transfected with HIF1α alone (Figure 2C).

Despite the reduced relevance of STAT3 and USF2 in HIF target gene activation in reporter gene assays, these results confirmed our previous findings that there is a specific functional interaction of USF2 with HIF2α on HIF2 target genes and of STAT3 with HIF1α on HIF1 target genes, and in addition we demonstrated that such specific functional interactions are maintained when both HIFα and STAT3 or USF2 are over-expressed.

### STAT3 increases the binding kinetics of HIF1α on the promoters of HIF1 target genes in RCC4T cells

**Figure 4:**
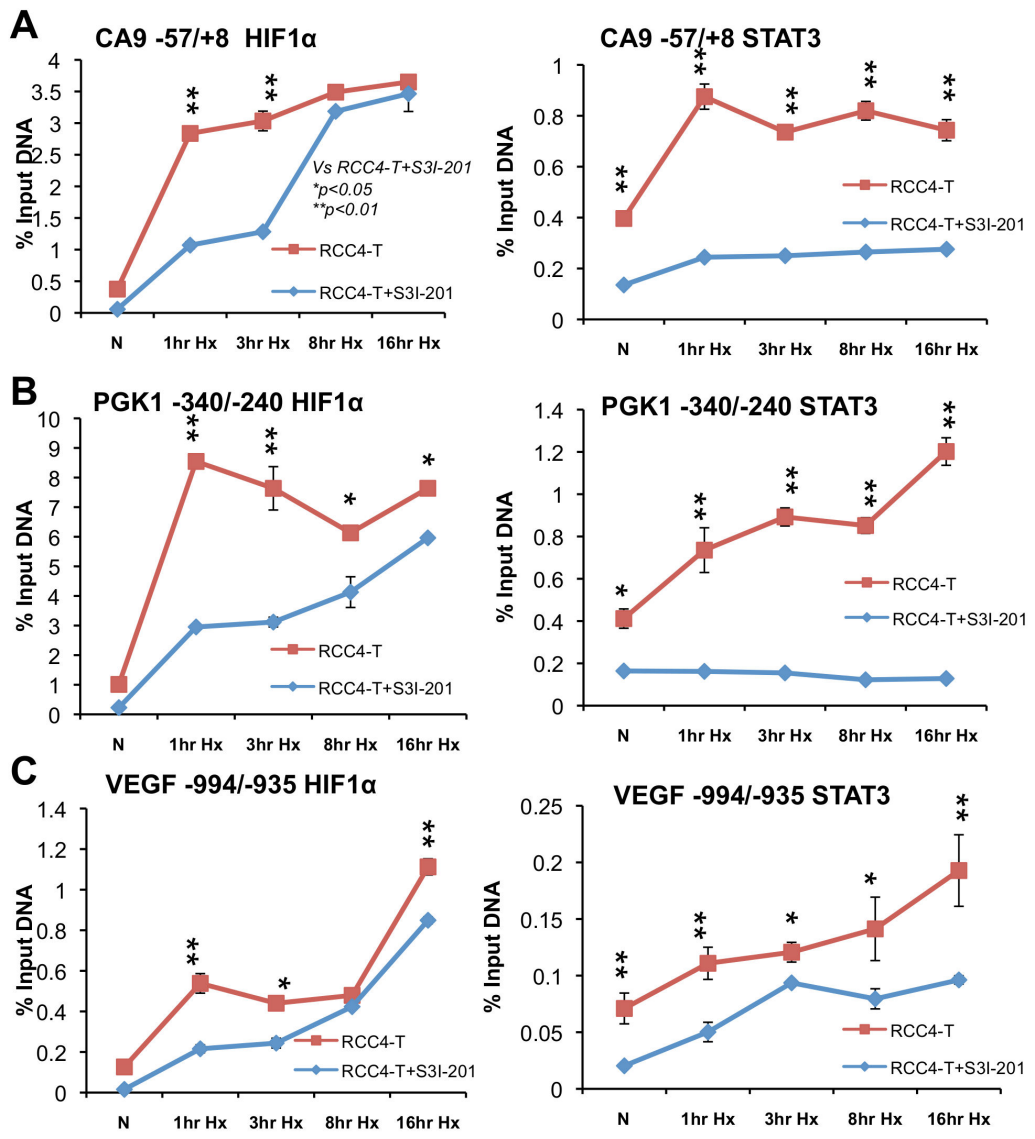
STAT3 increases the binding kinetics of HIF1α protein on its target gene promoters of CA9, PGK1 and VEGF in hypoxic RCC4T cells. Detection of HIF1α or STAT3 on the promoters of HIF1 target genes, *CA9* (**A**), *PGK1* (**B**) and *VEGF* (**C**) in RCC4T cells with or without STAT3 inhibitor S3I-201, cultured under normoxia or hypoxia for 1, 3, 8 and 16 hours. Results were expressed as % input DNA for easy appreciation of binding changes.

STAT3 and HIF1 cooperatively activate CA9 and PGK1 reporter genes while STAT3 itself exhibits only a weak or complete lack of activation of these reporters (Fig. 1). These findings prompted us to assess if STAT3 altered the binding kinetics of HIF1α on HIF1 target gene promoters and consequently promoted HIF1 to activate its target genes. To test this hypothesis, RCC4T cells that express constitutively active STAT3 [29] were cultured under hypoxia for 1, 3, 8 and 16 hours with or without STAT3 inhibitor. STAT3 and HIF1α binding on the promoters of the HIF1 target genes, *CA9*, *PGK1* and *VEGF* were assessed by ChIP. Hypoxia increased STAT3 binding on the promoters of *CA9, PGK1* and *VEGF* in RCC4T cells without STAT3 inhibitor (Fig. 4A–C, second column, red line) despite of constitutive Y705 phosphorylation of STAT3 in this cell line (see discussion). As expected, STAT3 inhibitor significantly reduced STAT3 binding to all three promoters (Fig. 4A–C, second column, blue lines). HIF1α binding on the *CA9* promoter was similar in 8 and 16-hour hypoxic RCC4T cells with or without STAT3 inhibitor while HIF1α binding on *PGK1* and *VEGF* promoters was slightly increased in 16-hour hypoxic RCC4T cells without STAT3 inhibitor than in RCC4T cells with STAT3 inhibitor (Fig. 4, first column), indicating STAT3 activity is not absolutely required for HIF1 binding to its target gene promoters under conditions of prolonged hypoxia. However, HIF1α binding to three HIF1 target gene promoters was much greater in 1 or 3-hour hypoxia treated cells without STAT3 inhibitor than in RCC4T cells with STAT3 inhibitor, indicating that STAT3 increased the binding kinetics of HIF1α to the promoters of HIF1 target genes in RCC4T cells (Fig. 4), a result consistent with the reported role of STAT3 in recruiting HIF1α to the VEGF promoter [35]. We do not think that increased detection of HIF1α on the promoters of HIF1 target genes in RCC4T is due to increased HIF1α stability, since we previously determined that STAT3 is not involved in HIF1α protein stability in RCC4T cells [36] although such function was reported in COS7 cells [37].

### N-TAD of HIF1α and HIF2α are important for specific functional interaction of HIF1α with STAT3 and of HIF2α with USF2 in reporter gene assays

**Figure 5:**
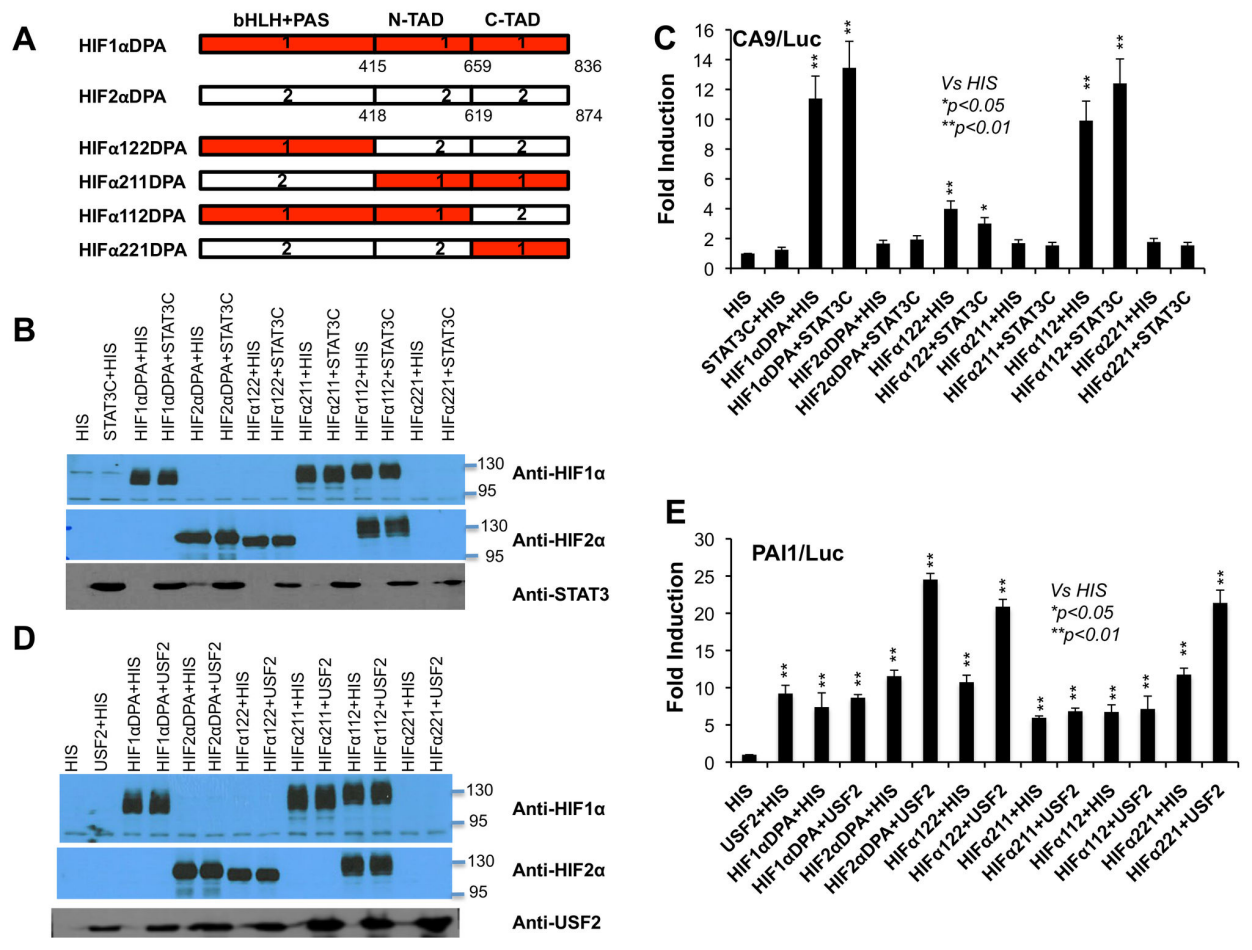
N-TADs of HIF1α or HIF2α is required for its functional interaction with STAT3 or USF2 to activate HIF1 or HIF2 target gene promoter. **A**) Schematic presentation of HIF1αDPA (Double Proline to Alanine), HIF2αDPA and HIF1α/HIF2α hybrid constructs. **B**) Western blot analysis of HIF1α, HIF2α, HIF hybrids and STAT3 to monitor the protein expression during CA9/Luc reporter gene assays. **C**) Activation of HIF1 target gene reporter, CA9/Luc, by the indicated plasmids. HIS is an empty vector that contains a histidine tag only. **D**) Western blot analysis of HIF1α, HIF2α, HIF hybrids and USF2 to monitor the protein expression during PAI1/Luc reporter gene assays. **E**) Activation of HIF2 target gene reporter, PAI1/Luc, by the indicated plasmids.

After establishing the functional interaction of HIF1α with STAT3 on the HIF1 target gene promoters, and of HIF2α with USF2 on the HIF2 target gene promoters, we wanted to determine the HIFα protein domains involved in functional interaction with STAT3 or USF2 protein. To test this, we used our previously generated HIF1α/HIF2α hybrid constructs in which domains from HIF1α were replaced by similar domains from HIF2α. For example, HIFα112 has HIF1α’s bHLH/PAS and N-TAD, but HIF2α’s C-TAD (Fig. 5A). HIF1α/HIF2α hybrid constructs (Fig. 5A) were transfected into HEK293T cells along with the STAT3C expression vector to test their cooperative activation of the HIF1 target gene promoter CA9/Luc (Fig. 5C) or with USF2 expression vector to test their cooperative activation of the HIF2 target gene promoter PAI1/Luc (Figure 5E). Like HIF1α, HIFα hybrid constructs (HIFα122 and HIFα112) containing the HIF1α bHLH+PAS domains were able to activate the CA9 reporter while HIFα hybrid constructs (HIFα211 and HIFα221) lacking the HIF1α bHLH+PAS domains only weakly activated the CA9 promoter (Figure 5C). This demonstrates that activation of a HIF1 target gene promoter CA9/Luc requires the HIF1α HLH/PAS domains, consistent with a previous report [22]. In addition, HIFα122 exhibited much lower activation of CA9/Luc than that of HIFα112, indicating strong activation of CA9/Luc also required the HIF1α N-TAD (Figure 5C). Consistent with results in Figure 1D, STAT3C was able to enhance activation of CA9/Luc by HIF1αDPA, but not by HIF2αDPA (Figure 5C). Interestingly, STAT3C also promoted activation of CA9/Luc by the HIFα112 hybrid protein that possessed the HIF1α bHLH+PAS and N-TAD domains (Figure 5C, HIFα112) but did not cooperate with the HIFα122 hybrid protein although HIFα122 also activated the CA9/Luc reporter (Figure 5C, HIFα122), indicating the importance of the HIF1α N-TAD in the functional interaction between HIF1α and STAT3. Importantly, HIF1α, HIF2α and HIF1/HIF2 hybrid constructs expressed similar amounts of proteins as detected by anti-HIF1α or anti-HIF2α antibodies; however, the HIFα221 protein was not detected by either antibody (Figure 5B).

The HIF2 target promoter PAI1 was activated by HIF1α, HIF2α and all HIFα hybrid constructs (Fig. 5E). Interestingly, cooperative activation of PAI1/Luc was only observed for USF2 with HIF2α and HIFα hybrids containing the HIF2α N-TAD (HIFα122 and HIFα221), but not with HIF1α and HIFα hybrids containing the HIF1α N-TAD (HIFα211 and HIFα112) (Fig. 5E). These results demonstrate that the HIF2α N-TAD is required for functional cooperation with USF2 in activating the HIF2 target promoter PAI1 (Fig. 5E). Additionally, the low activation of the PAI reporter by HIF1, HIF1+USF2, or others was not due to low protein expression (Fig. 5D). In conclusion, these experiments demonstrated that the N-TADs of HIF1α or HIF2α are required for functional interaction with STAT3 or USF2 protein.

### The HIF1α/STAT3 physical interaction requires the HIF1α bHLH and PAS domains, but not the HIF1α *N-*TAD

**Figure 6:**
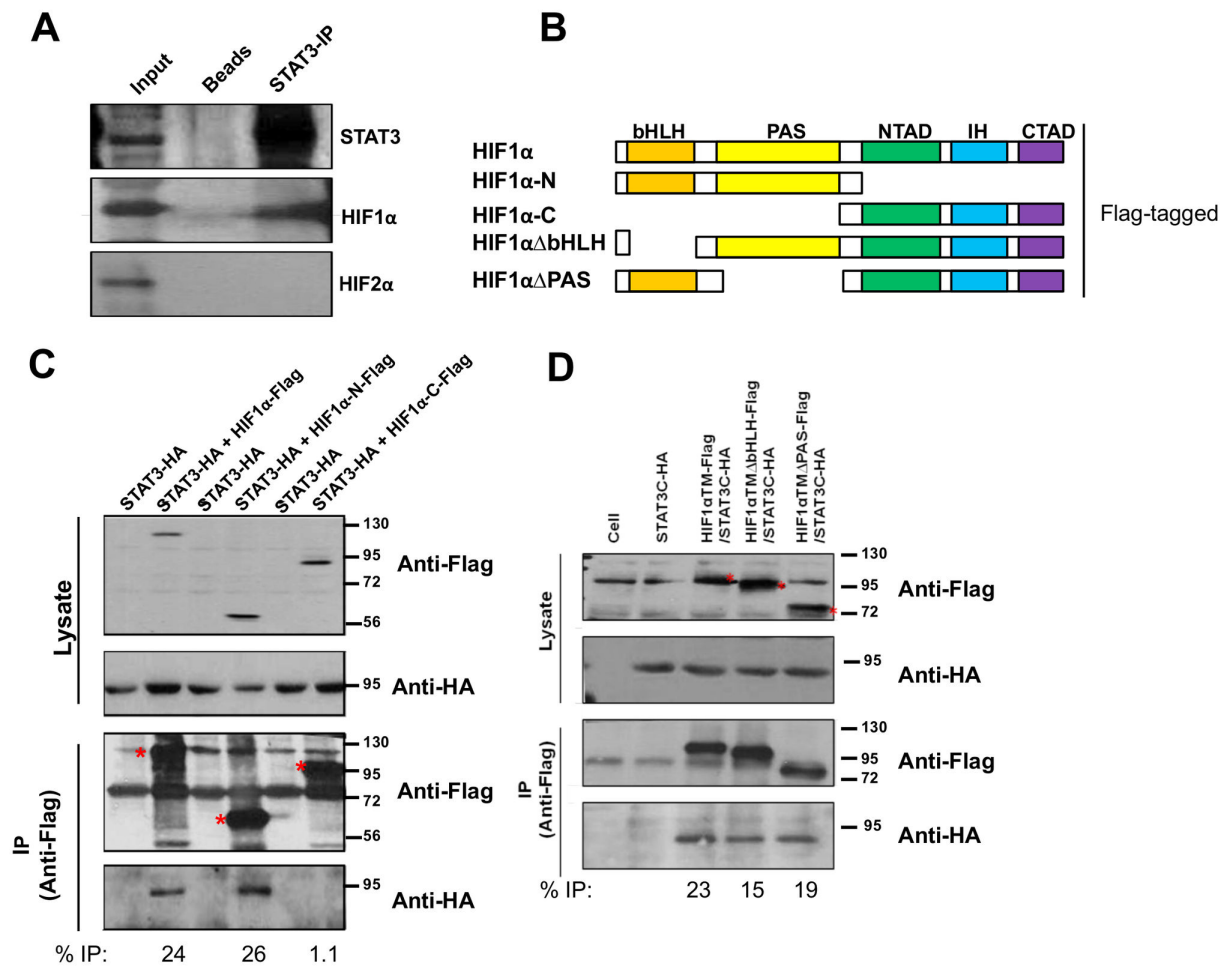
The HIF1α/STAT3 physical interaction requires the HIF1α bHLH and PAS domains. **A**) WB detection of STAT3, HIF1α and HIF2α in cell lysate (input) prepared from normoxic RCC4 cells, in precipitated materials from the protein A/protein G beads and pre-immuno serum (beads) or in precipitated materials from STAT3 antibody and protein A/protein G beads (STAT3-IP). **B**) Schematic presentation of Flag-tagged full-length (HIF1α), N-terminal (HIF1α-N) or C-terminal (HIF1α-C) halves of HIF1αTM or HIF1αTM-Flag deletion of bHLH (HIF1α∆bHLH) or PAS domains (HIF1α∆PAS). **C**) Anti-Flag or anti-HA WB detection of Flag-tagged HIF1α or HA-tagged STAT3C protein in cell lysates (Lysates) or in anti-Flag beads precipitated materials (IP). The red stars indicate the precipitated FL, HIF1α-N and HIF1α-C proteins. The percentage of co-precipitated STAT3 protein was calculated by dividing the density of the "anti-HA IP" band by the density of the "anti-Flag IP" bands to control for the different amount of precipitated Flag-tagged HIFα protein. Similar calculation was performed for Figure 6D and Figure 7C-D. D) Anti-Flag or anti-HA WB detection of Flag-tagged HIF1α or HA-tagged STAT3C protein in cell lysates (Lysates) or in anti-Flag beads precipitated materials (IP).

After observing that the N-TAD of HIF1α or HIF2α was required for HIF’s functional cooperation with another transcription factor, we reasoned that the HIFα N-TADs might be necessary to mediate a specific physical association with their respective transcription factor. To test this hypothesis, we first validated a specific physical interaction between the HIF1α and STAT3 proteins by showing that immuno-precipitation of endogenous STAT3 in RCC4 cells co-precipitated HIF1α, but not HIF2α, despite detectable amounts of both HIFα proteins in the nuclear extracts (Figure 6A). To identify which region(s) of the HIF1α protein were required for its physical interaction with STAT3, we generated Flag-tagged HIF1αTM deletion mutants. The HIF1α-N construct contained the N-terminal half of HIF1αTM including the bHLH and PAS domains while the HIF1α-C contained the N-TAD, IH and C-TAD domains of HIF1αTM (Figure 6B). Flag-tagged FL (full-length) HIF1αTM or its deletion mutants were transfected into HEK293T cells with HA-tagged constitutively active STAT3C and lysates were subjected to anti-Flag immuno-precipitation. Analysis of co-precipitated HA-tagged STAT3C protein revealed that STAT3C physically interacted with full-length HIF1αTM and HIF1α-N protein, but not with HIF1α-C protein, suggesting that the HIF1α’s bHLH and PAS domains were required for the physical interaction with STAT3 (Figure 6C).

To determine which sub-region of HIF1α is required for the physical interaction with STAT3, we deleted bHLH or PAS domains from full-length HIF1α (Figure 6B). Pull-down of HIF1α∆bHLH or HIF1α∆PAS also co-precipitated a significant amount of HA-tagged STAT3 (Figure 6D), indicating that the HIF1α bHLH or PAS domain alone was sufficient for physical interaction with STAT3.

### The HIF2α/USF2 physical interaction requires the C-TAD and N-TAD of the HIF2α protein

**Figure 7:**
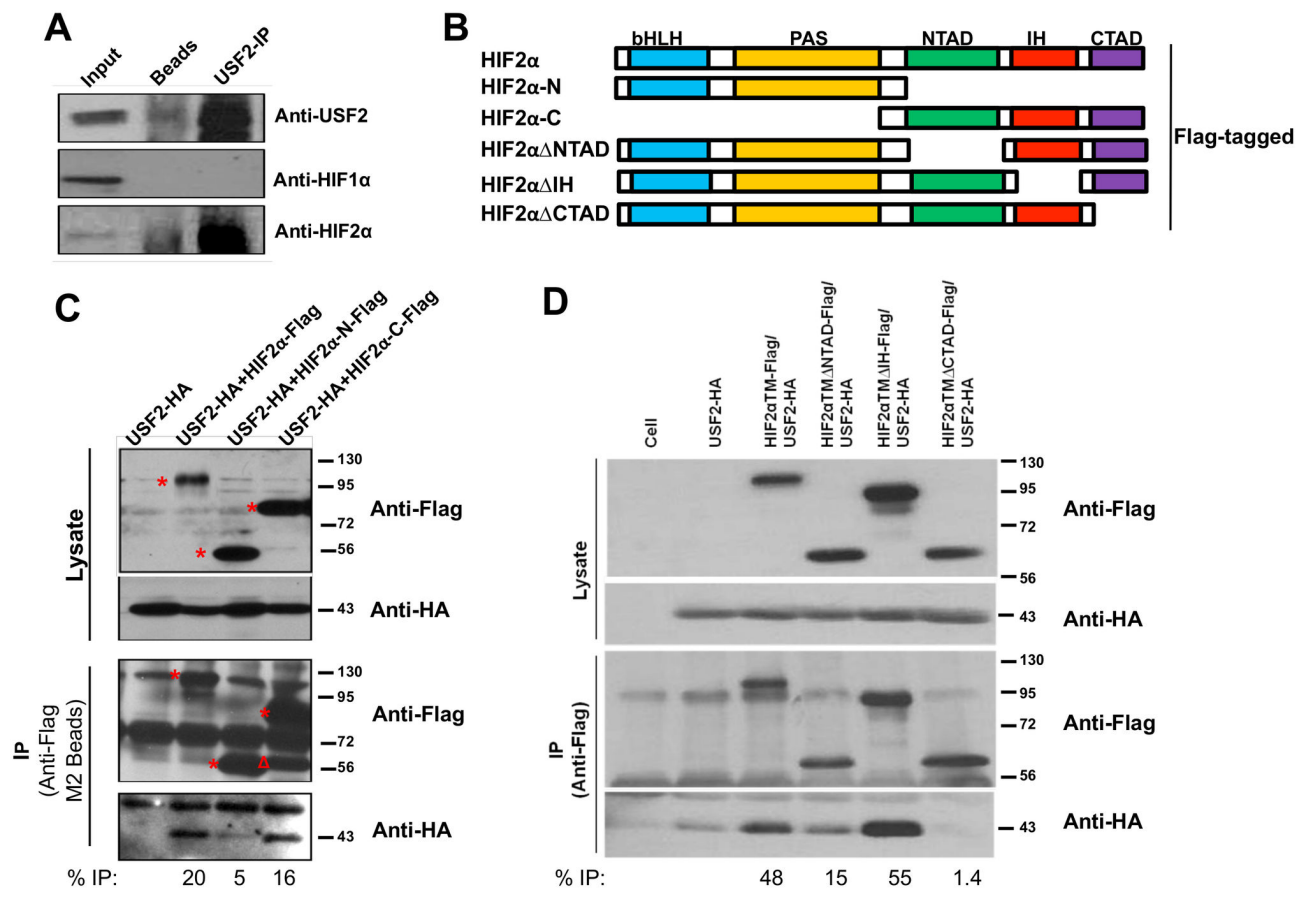
The HIF2α/USF2 physical interaction requires the C-TAD and N-TAD of the HIF2α protein. **A**) WB detection of USF2, HIF1α and HIF2α in RCC4 cell lysate (input), in precipitated materials by the protein A/protein G beads and pre-immuno serum (beads) or in precipitated materials by USF2 antibody and protein A/protein G beads (USF2-IP). **B**) Schematic presentation of Flag-tagged full-length (HIF2α), N-terminal (HIF2α-N) or C-terminal (HIF2α-C) halves of HIF2αTM or HIF2α deletion of N-TAD (HIF2α∆NTAD), IH (HIF2α∆IH) or C-TAD (HIF2α∆CTAD). **C**) Anti-Flag or anti-HA WB detection of Flag-tagged HIF2α or HA-tagged USF2 protein in cell lysates (Lysates) or in anti-Flag beads precipitated materials (IP). The red starts indicated the positions of HIF2αFL, HIF2α-N and HIF2α-C proteins. The Δ labeled band in USF2+HIF2-C lane was consistently observed, likely expressed from downstream ATG of HIF2α cDNA. **D**) Anti-Flag or anti-HA WB detection of Flag-tagged HIF2α or HA-tagged USF2 protein in cell lysates (Lysates) or in anti-Flag beads precipitated materials (IP). The background signals in USF2-HA only lane was deducted from the “anti-HA IP’ signal to calculate the % IP.

We also confirmed the physical interaction of HIF2α with USF2 that we previously reported in hypoxic Hep3B cells [29] now in RCC4 cells by showing that immuno-precipitation of endogenous USF2 protein co-precipitated HIF2α, but not HIF1α protein (Fig. 7A). To determine which domains of HIF2α mediate its interaction with USF2 protein, we first generated Flag-tagged HIF2αTM deletion mutants: HIF2α-N that contained the HIF2α bHLH and PAS domains, and HIF2α-C that contained the N-TAD, IH, and C-TAD domains (Fig. 7B). The Flag-tagged FL HIF2α or its deletion mutants were co-transfected with HA-tagged-USF2 in HEK293T cells and lysates were subjected to anti-Flag immuno-precipitation. Analysis of co-precipitated HA protein revealed that USF2 physically interacted with full-length HIF2αTM and HIF2α-C protein, but its interaction with HIF2α-N protein was much reduced (Fig. 7C), suggesting that the HIF2α protein C-terminal half was required for the HIF2α/USF2 physical interaction.

To determine which sub-region of HIF2α C-terminal half is required for the physical interaction with USF2, we deleted N-TAD, IH or C-TAD domain from full-length HIF2α (Fig. 7B). HIF2α∆C-TAD was not able to co-precipitate USF2 while HIF2α∆N-TAD reduced its interaction with USF2 to about 30% capability of the full-length HIF2α (Fig. 7D). However, HIF2α∆IH maintained its full ability to associate with USF2 (Fig. 7D). These results indicated that both N-TAD and particularly the C-TAD of HIF2α are critical for HIF2α/USF2 physical interaction.

### Addition of USF2 binding sites, but not a change or addition of HIF binding site (HBS) increases the basal activity and HIF1/HIF2 responsiveness of the CA9 reporter in a position dependent manner

The above data suggested that specific binding of STAT3 or USF2 to the promoters of HIF1 or HIF2 target genes, plus specific functional and physical interaction of STAT3 with HIF1α or USF2 with HIF2α on the promoters of HIF1 or HIF2 target genes could contribute to HIF target gene specificity. To directly test the importance of STAT3 or USF2 binding sites in determining HIF target gene specificity, we wanted to see if addition of USF2 binding sites into a HIF1 target gene promoter could change the promoter’s response to HIF activation. We focused on USF2 and USF2 binding sites since STAT3 itself is a weak activator in the reporter gene assays. At the same time, we wanted to test if the HBS in a HIF1 target gene is truly interchangeable with an HBS from a HIF2 target gene, as no study has been performed to directly compare the possible functional difference between HBSs from HIF1 and HIF2 target genes. We selected CA9 as a model HIF1 target gene for the following reasons. 1. *CA9* is a HIF1 target gene in all cell types assessed [12,22,23,28,29]; 2. CA9 is one of the few HIF1 target genes whose promoters are exclusively bound by HIF1α, not HIF2α protein in MCF-7 cells [23]. We selected PAI1 as a HIF2 target gene since PAI1 is a well-established HIF2 target gene whose promoter contains a single functional HBS at -191 and several functional USF2 binding sites [29,38].

**Figure 8:**
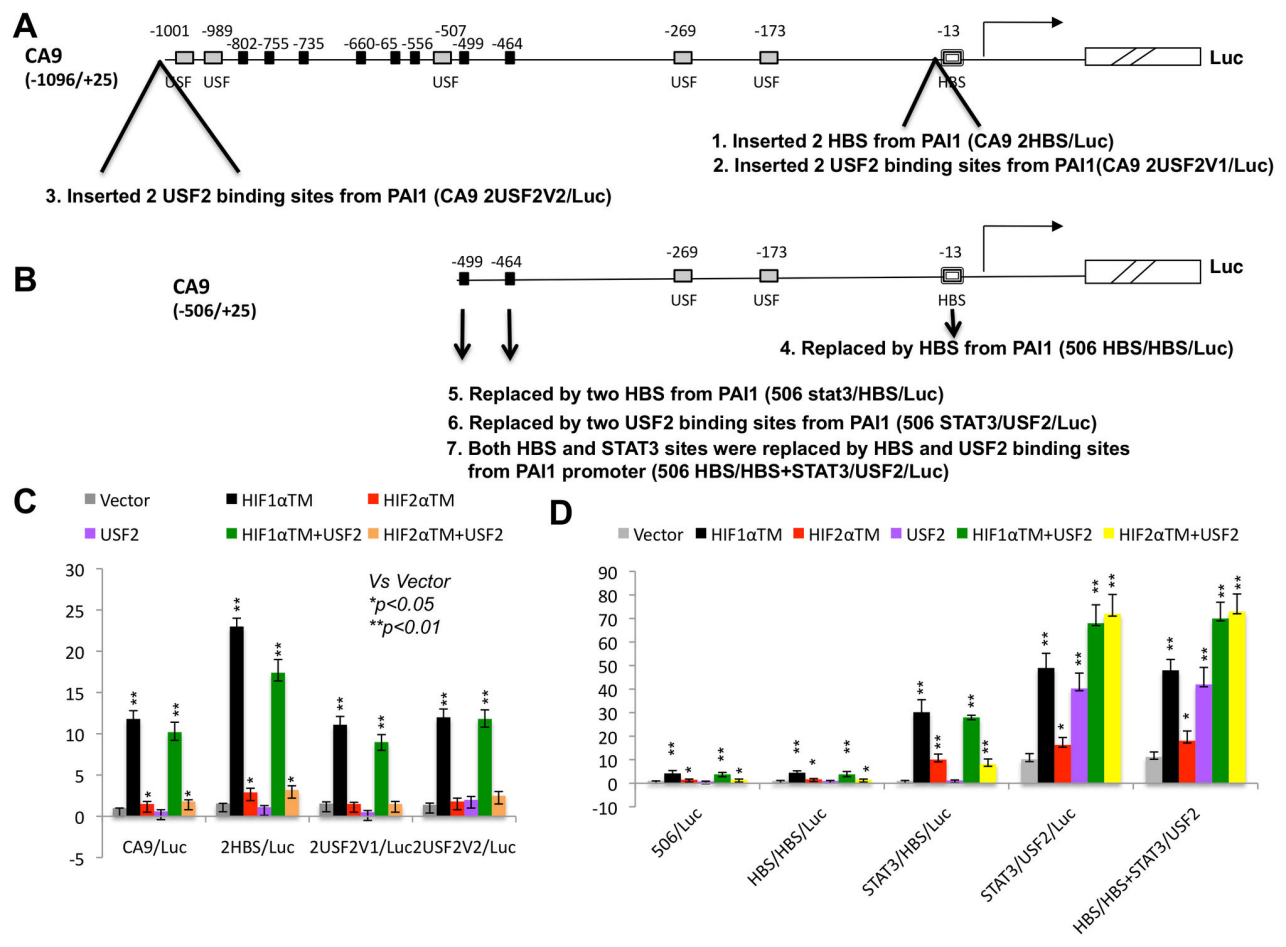
The role of HIF and USF2 binding sites on HIF target gene specificity. **A**) Schematic presentation of the *CA9* promoters, a HIF1 target gene. Construct 1 was generated by inserting 2 copies of -191 HBS of PAI1 promoter, a HIF2 target gene near the -13 HBS of CA9 promoter. Constructs 2 and 3 were generated by inserting -684 and -565 USF2 binding sites of PAI1 promoter near the -13 HBS (construct 2) or near -1001 of CA9 promoter (construct 3). **B**) Schematic presentation of a shorter version of *CA9* promoters (-506/+25). Construct 4 was generated by replacing the original -13 HBS with -191 HBS of PAI1 promoter. Constructs 5 and 6 were generated by replacing the STAT3 binding sites at -499 and -464 of CA9 promoter with -191 HBS (construct 5) or -684 and -565 USF2 binding sites (construct 6) from the PAI1 promoter. Construct 7 was made by replaced -13 HBS and -499 and -464 STAT3 binding sites in the CA9 promoter with HBS and USF2 binding sites from PAI1 promoter. **C**) Fold of induction of CA9/Luc reporters (-1096/+25) activated by the indicated plasmids. **D**) Fold of induction of CA9/Luc reporters (-506/+25) activated by the indicated plasmids. The same activators used in Figure 1 were used for experiments here.

We first inserted two copies of the -191 HBS (the 6 bp core plus flanking 6 bp from both sides, total 18 bp) from the PAI1 promoter into the CA9/Luc reporter near the -13 HBS (Fig. 8A, construct 1) to mimic the artificial HIF target promoters that contain multiple HBSs. Interestingly, the CA9 2HBS/Luc reporter exhibited about 2-fold increased activation by both HIF1 and HIF2 (Fig. 8C). However, like the parental CA9/Luc, the 2HBS/Luc reporter still exhibited much stronger activation by HIF1 (23 fold) than by HIF2 (2.9 fold) (Fig. 8C), no activation by USF2 and reduced activation by HIF1α+USF2 (17.4 fold) than by HIF1α only (23 fold). We then inserted the -684 and -565 USF2 binding sites (18 bp each) from the PAI1 promoter (Fig. 1B) near the -13 HBS of CA9/Luc (Fig. 8A, construct 2). Surprisingly, CA9 2USF2V1/Luc was not activated by USF2 (Fig. 8C) although these USF2 sites in the PAI1 promoter were demonstrated as functional [29,38]. Additionally, in comparison with CA9/Luc, CA9 2USF2V1/Luc also was similarly and strongly activated by HIF1 (11.1 fold) and weakly activated by HIF2 (1.5 fold) (Fig. 8C). We then inserted the two same USF2 binding sites around -1001 of the CA9 promoter (Fig. 8A, construct 3). Again, this construct was resistant to USF2 activation and exhibited identical properties of the parental reporter of CA9/Luc with stronger response to HIF1 (12 fold) or weak response to HIF2 (1.8 fold) activation (Fig. 8C).

To further study the possible difference of HBSs in the promoters of *CA9* and *PAI1* and the role of USF2 binding sites for HIF2’s ability to activate a target gene, we deleted the -1096/-506 region from CA9/Luc and made a -506/+25 CA9/Luc reporter that contained only two potential STAT3 binding sites (Fig. 8B). We found that this -506/+25 CA9/Luc reporter was weakly activated by STAT3C alone (1.5 fold) and there was an additive activation by HIF1 and STAT3C (Fig. S1B). However, deletion of a region from -1096 to -171 led to loss of activation by STAT3C and loss of additive activation by HIF1α+STAT3C (Fig. S1B, CA9 -171/+25), indicating that the STAT3 binding sites at -499 and -464 of the CA9 promoter were active. For the CA9 -506/+25/Luc reporter, we first replaced the original -13 HBS in the CA9 promoter with the -191 HBS (18 bp) from the PAI1 promoter (Fig. 8B, construct 4). In comparison to the parental CA9 -506/+25/Luc reporter, the -506/+25 HBS/HBS/Luc reporter exhibited an identical response to activation of HIF (4.2 fold by HIF1 and 1.7 fold by HIF2) or USF2 (no activation) individually or in combination (Fig. 8D). We then replaced the two STAT3 binding sites at -499 and -464 with two copies of the 18 bp -191 HBS from PAI1 promoter (Fig. 8B, construct 5). The -506/+25 STAT3/HBS reporter exhibited similarly increased activation by both HIF1 (30.2 fold with 7.2 times of increasing) and HIF2 (10.1 fold with 5.9 times of increasing). These results indicated that increased number of HBSs enhances the response of the reporter to both HIF1 and HIF2 activation. We then replaced the -499 and -464 STAT3 binding sites in the CA9 promoter with the -684 and -565 USF2 binding sites from the PAI1 promoter (Fig. 8B, construct 6). Interestingly, this -506/+25 STAT3/USF2 reporter exhibited several interesting new properties in comparison with the parental-506/+25/Luc reporter. First, this reporter exhibited significantly increased basal activity (10.2 fold, relative to CA9 -506/+26/Luc by vector). Second, USF2 activated this reporter (4.0 fold, relative to the same reporter by vector). Third, the reporter was better activated by HIF1+USF2 (6.8 fold) than by HIF1 (4.9 fold) or USF2 alone (4.0 fold). Most importantly this reporter was additively activated by HIF2+USF2 (7.2 fold by HIF2+USF2, 1.7 fold by HIF2 and 4.0 fold by USF2) (Fig. 8D). These data confirmed the importance of USF2 binding sites for basal activity of the reporter gene and for HIF2 to activate HIF2 target genes. However, this reporter still maintained its stronger activation by HIF1 alone (4.9 fold) and weaker activation by HIF2 alone (1.63 fold) (Fig. 8D), suggesting that functional USF2 binding sites alone contribute significantly, but are not sufficient to change a HIF1 target gene promoter to a HIF2 target gene promoter. To additionally test the possible contribution of the -13 HBS of the CA9 promoter to HIF1 target gene identity, we then replaced the -13 HBS in the CA9 promoter with the -191 HBS from the PAI1 promoter as well as replaced the STAT3 binding sites in the CA9 promoter with the two USF2 binding sites from the PAI1 promoter. This -506/+25 HBS/HBS+STAT3/USF2 reporter (construct 7) behaved identical to the 506 STAT3/USF2/Luc (Fig. 8D), further confirming the interchangeability of HBSs from a HIF1 or HIF2 target gene.

### Pol II/HIF1α or Pol II/HIF2α transcriptional complexes are detected on the promoters of HIF1 and HIF2 target genes respectively in a STAT3 or USF2 dependent manner

**Figure 9:**
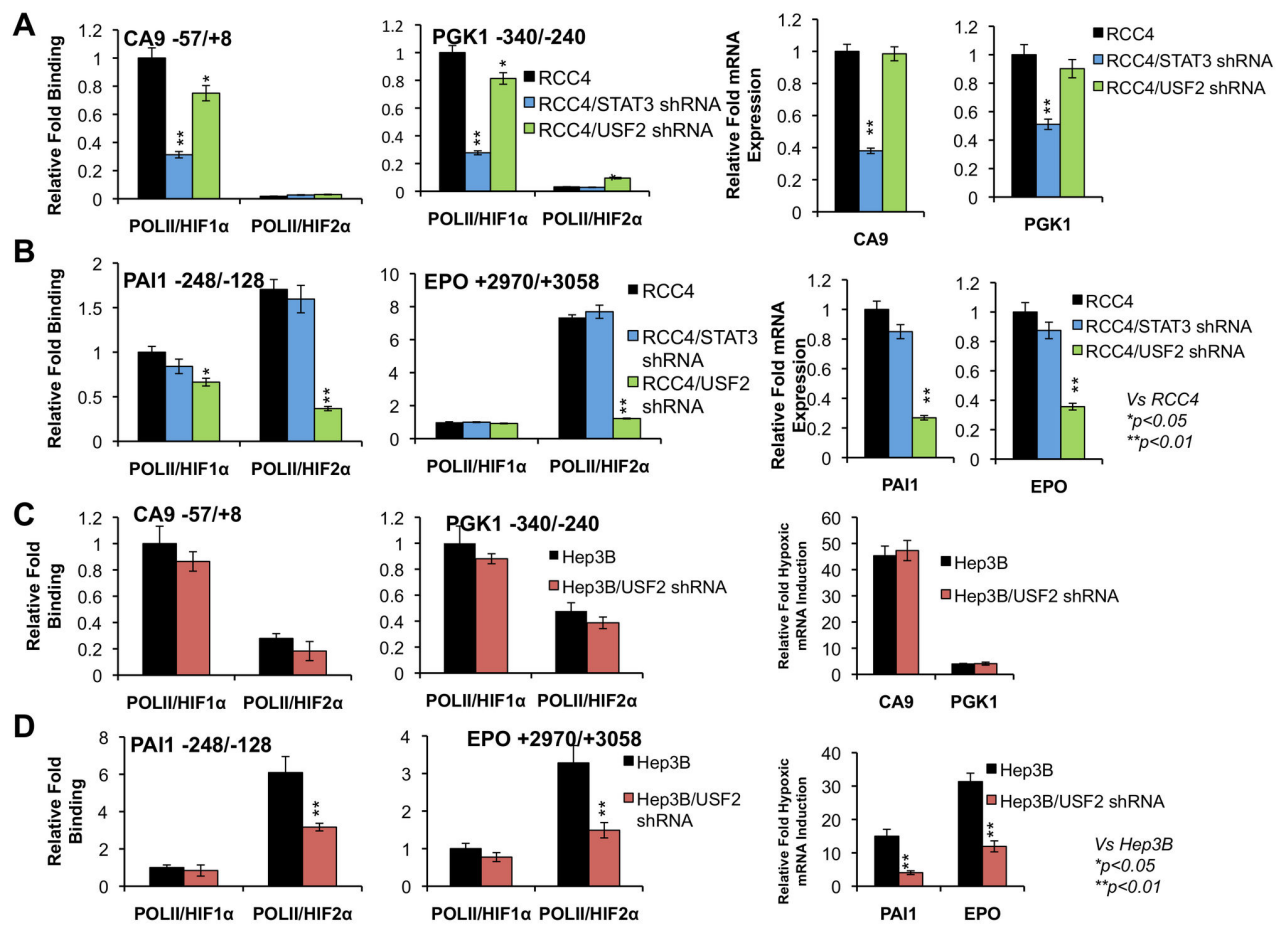
HIF1 or HIF2 target gene promoters/enhancers are bound by distinct HIF1α/Pol II or HIF2α/Pol II transcriptional complexes and formation of these transcriptional complexes depends on STAT3 or USF2 activity. Sonicated chromatin from normoxic RCC4, RCC4/STAT3 shRNA, RCC4/USF2 shRNA or hypoxic Hep3B cells or Hep3B/USF2 shRNA cell was subjected to anti-Pol II ChIP, the precipitated protein/DNA complexes were then subjected to a secondary ChIP using HIF1α or HIF2α antibodies. Precipitated DNA was analyzed for HIF target gene promoter and results were displayed as percent of relative fold of binding. **A**) Detection of Pol II/HIF1α or Pol II/HIF2α complexes on the promoters of HIF1 target genes, *CA9* and *PGK1* (two left columns) and mRNA levels of CA9 and PGK1 in normoxic RCC4, RCC4/STAT3 shRNA and RCC4/ USF2 shRNA cells (two right columns). **B**) Detection of Pol II/HIF1α or Pol II/HIF2α complexes on the promoter or enhancer of HIF2 target genes, *PAI1* and *EPO* (two left columns) and mRNA levels of PAI-1 and EPO in normoxic RCC4, RCC4/STAT3 shRNA and RCC4/ USF2 shRNA cells (two right columns). **C**) Detection of Pol II/HIF1α or Pol II/HIF2α complexes on the promoters of HIF1 target genes, *CA9* and *PGK1* and fold of induction of CA9 and PGK1 gene expression in hypoxic Hep3B and Hep3B/USF2 shRNA cells. **D**) Detection of Pol II/HIF1α or Pol II/HIF2α complexes on the promoter or enhancer of HIF2 target genes, *PAI1* and *EPO* and fold of induction of PAI1 and EPO in hypoxic Hep3B and Hep3B/USF2 shRNA cells.

Based on specific binding of STAT3 and USF2 to HIF1 and HIF2 target gene promoters respectively and specific physical interaction of HIF1α with STAT3 on HIF1 target gene promoters or HIF2 with USF2 on HIF2 target gene promoters, we reasoned that while both HIF1α and HIF2α bind to HIF target promoters, only one HIF subunit might be associated with the functional, transcription-competent RNA polymerase II-containing complex on HIF gene promoters. We therefore sought to determine if the functional HIFα subunit was more closely associated with RNA polymerase II on the promoters of its target genes. We conducted ChIP/ReChIP experiments in RCC4 cells, a cell line in which STAT3 and USF2 are specifically required for HIF1α and HIF2α respectively [28,29]. Chromatin isolated from RCC4 cells was first subjected to traditional ChIP using an antibody recognizing the largest subunit of the RNA polymerase II complex (Pol II). The precipitated protein/DNA complexes were then eluted and subjected to a second round of ChIP using antibodies against HIF1α or HIF2α. Interestingly, RNA Pol II was found to be mainly associated with HIF1α protein compared with HIF2α protein on the promoters of HIF1 target genes, *CA9* and *PGK1* (Fig. 9A). However, RNA Pol II was found to be associated with HIF2α protein compared with HIF1α protein on the promoters of HIF2 target genes, *PAI1* and *EPO* (Fig. 9B). To test the role of STAT3 and USF2 in the formation of the Pol II containing complex, similar experiments were conducted in RCC4 cells in which STAT3 or USF2 were stably knocked down. Consistent with previous results [28,29], STAT3 knockdown reduced expression of HIF1 target genes *CA9* and *PGK1* (Fig. 9A, third and fourth columns) while USF2 knockdown reduced levels of HIF2 target genes *PAI1* and *EPO* (Fig. 9B, third and fourth columns). Interestingly, RCC4/STAT3 shRNA cells exhibited reduced formation of the Pol II/HIF1α complex on the HIF1 target gene promoters CA9 and PGK1 (Fig. 9A), but not on the HIF2 target gene promoters PAI1 and EPO (Fig. 9B). Additionally, RCC4/USF2 shRNA cells exhibited significantly reduced formation of the Pol II/HIF2α complex on the HIF2 target genes PAI1 and EPO (Figure 9B), and also slightly reduced formation of the Pol II/HIF1α complex on the HIF1 target gene promoters of CA9 and PGK1 (Figure 9A).

We also performed similar experiments in hypoxic Hep3B cells or Hep3B cells in which USF2 is stably knocked down (Fig. 9C-D). RNA Pol II was found to be associated mainly with HIF1α protein compared with HIF2α protein on the promoters of HIF1 target genes, *CA9* and *PGK1* (Fig. 9C). However, RNA Pol II was found to be associated much more with HIF2α protein compared with HIF1α protein on the promoters of HIF2 target genes, *PAI1* and *EPO* (Fig. 9D). Knockdown of USF2 reduced Pol II/HIF2α on the promoters of HIF2 target genes *PAI1* and *EPO* (Fig. 9D), but not on HIF1 target genes *CA9* and *PGK1* (Fig. 9C). Functionally, USF2 knockdown only reduced hypoxic induction of HIF2 target genes (Figure 9D), but not HIF1 target genes (Figure 9C), a result in agreement with our previous findings [29].

These results demonstrate that although HIF target promoters may be bound by both HIF1α and HIF2α protein, only one HIFα protein is associated with a functional, Pol II-containing transcriptional complex. Most importantly, STAT3 and USF2 play significant roles in the formation of Pol II/HIF1α or Pol II/HIF2α complexes on the HIF1 or HIF2 target gene promoters.

## Discussion

The HIF1 and HIF2-mediated transcriptional responses are important for solid tumor progression. Although highly similar [7–11], HIF1 and HIF2 activate unique and common target genes, a phenomenon found in cultured cells when cells were targeted with siRNA or shRNA to reduce endogenous HIF1α or HIF2α mRNAs or in animal models possessing specific HIFα gene knockout [12–21]. However, over-expression of normoxia-active HIFα in normoxia-cultured cells disrupts HIF target gene specificity. These data indicate that the binding of HIF1 and HIF2 protein to HIF target gene promoters is potentially indiscriminate.

In this report, we first confirmed our previous results that STAT3 and USF2 bind to the promoters of HIF1 or HIF2 target genes respectively [28,29] (Fig. 3). Additionally, we uncovered that STAT3 or USF2 does not bind to the promoters of HIF2 or HIF1 target genes respectively (Fig. 3). Furthermore, we showed here, for the first time, that even over-expressed USF2 selectively promotes HIF2 target gene activation in functional assays (Fig. 1 and 2). Thus, unlike HIF1 and HIF2, STAT3 and USF2 exhibit specific binding to the promoters of HIF1 or HIF2 target genes.

We then confirmed a functional interaction of HIF2α with USF2 on HIF2 target genes, and STAT3 with HIF1 on HIF1 target genes in experimental conditions where HIF, STAT3 and USF2 are over-expressed (Fig. 1 and 2). However, we observed that USF2 is less important for HIF2 target gene promoter activation in over-expression assays than for endogenous gene activation; this is particularly true for STAT3 activation of HIF target gene promoters (Fig. 1 and 2). For example, although we previously showed that inhibiting STAT3 activity reduces hypoxic induction of HIF1 target genes by 50% in RCC4 and MDA-MB-231 cells [28], we found here that STAT3 co-transfection only moderately increases HIF1’s ability to activate HIF1 target genes in reporter gene assays. We speculate that over-expressed HIF protein may bind to imperfect HBS sequences, such as E-boxes (CANNTG) on the HIF target promoters, which may initiate transcription independent of HIF transcription partners (e.g STAT3 and USF2) that are normally required in a physiological setting.

The specific functional interaction observed in our over-expression system established a foundation for us to determine which protein domains of HIF1α and HIF2α are involved in their functional interaction with STAT3 and USF2. We demonstrate here for the first time that the N-TADs of HIF1α and HIF2α are required for functional cooperation with their respective co-transcriptional activator proteins in the activation of HIF1 or HIF2 target genes, supporting the previously reported results that the N-TADs of HIFα are important in determining HIF target gene specificity [12,22]. In contrast to HIF2α in which only the N-TAD is required for its functional interaction with USF2, the HIF1α HLH and PAS domains are also required for functional interaction with STAT3, which might explain this previously reported phenomenon that HIF1α HLH and PAS domains are also important for HIF target gene specificity [22]. Consistent with functional data, HIF1α HLH and PAS domains are important for physical interaction with STAT3 while the HIF2α N-TAD is also involved in HIF2α/USF2 physical interaction.

We then tested the importance of HBSs and USF2 binding sites in determining HIF target gene specificity. Although multiple studies have suggested that HIF1 and HIF2 bind to HIF target gene promoters indiscriminately, a recent study found that the promoter of the HIF1 target gene, *CA9*, is only associated with HIF1 protein [23]. We, for the first time, provide evidence that the HBS of the CA9 promoter is not functionally different from the HBS of a HIF2 target gene (*PAI1*) in reporter gene assays. We show that replacement of the CA9 HBS by the PAI1 HBS does not change the response of this reporter to HIF or USF2 activation (Fig. 8D, comparing HBS/HBS/Luc with CA9 -56/+25/Luc or STAT3/USF2/Luc with STAT3/USF2+HBS/HBS/Luc). Also, insertion of the PAI1 HBS into the CA9 promoter does not change the fact that these new reporters are still activated more strongly by HIF1 than HIF2 (Figure 8C, comparing 2HBS/Luc with CA9/Luc. Figure 8D, comparing STAT3/HBS/Luc with 506/Luc).

We then tested the importance of USF2 binding sites in HIF2’s ability to activate a reporter gene (Fig. 8). These experiments generated interesting findings as described below(1). The activity of USF2 binding sites is highly position or context dependent. We found that -1096/+25 CA9 reporter containing two USF2 binding sites at -13 or -1001 regions did not respond to USF2 activation (Fig. 8C). These data could also be used to explain why HIF1 target genes are not activated by USF2 although these HIF1 target gene promoters contain similar numbers of potential USF2 binding sites as HIF2 target promoters(2). Functional USF2 binding sites are very important for basal activity of the promoter. We found that replacing two STAT3 binding sites with USF2 binding sites increases the basal activity of the CA9 reporter by 10 fold (Fig. 8D, STAT3/USF2/Luc), further confirming our previous results that USF2 binding sites are very important for basal activity of PAI1 promoter, a HIF2 target gene [29]. (3) Although HIF1+USF2 activate the -506/+25 STAT3/USF2 reporter stronger than HIF1 or USF2 alone (Fig. 8D), this result is opposite to what we observed in native HIF1 target gene promoters of CA9 and PGK1 in which co-transfection of USF2 inhibits activation of these promoters by HIF1 (Fig. 1D and 2B). This result is also different from what we observed in native HIF2 target gene promoters of PAI1 and EPO in which USF2 does not change HIF1’s activation of these promoters (Figs. 1 and 2). We speculate that since either HIF1 or USF2 activates the -506/+25 STAT3/USF2 reporter, HIF1 and USF2 might activate reporter gene expression independent of each other’s function in initiating transcription(4). USF2 binding sites are critical for HIF2 to activate HIF2 target gene. The activation of -506/+25 STAT3/USF2 reporter by HIF2+USF2 is additive while activation of this reporter by HIF1α+USF2 is not. These results further support a specific functional interaction between HIF2 and USF2(5). Although the -506/+25 STAT3/USF2 reporter could be strongly activated by USF2, this reporter is still activated by HIF1 better than HIF2 (Fig 8D) while native HIF2 target gene promoters such as PAI1 and EPO exhibit better activation by HIF2 than HIF1 (Fig. 1E and 2C). Thus, besides USF2 binding sites, other sites via their associated transcription factors also contribute to HIF target gene specificity. Indeed, several other HIF1α or HIF2α specific co-transcriptional factors such as ETS1, ELK and NEMO are reported [26,39–42].

Finally, we determined the role of STAT3 and USF2 in forming RNA-polymerase-containing transcription complexes on the promoters of HIF1 or HIF2 target genes. Although we previously demonstrated interaction of Pol II with STAT3 and HIF1α with STAT3 on the promoters of HIF1 target genes, and Pol II with USF2 and HIF2α with USF2 on the promoters of HIF2 target genes [28,29], we reported here for the first time that Pol II on the HIF1 target gene promoters is mainly associated with HIF1α while Pol II on the HIF2 target gene promoters is mainly associated with HIF2α. Importantly, STAT3 or USF2 plays an important role in the formation of Pol II/HIF1α or Pol II/HIF2α complexes on the promoters of HIF1 or HIF2 target genes (Fig 9). However, we found that knockdown of USF2 in RCC4 cells, but not in hypoxic Hep3B cells, also slightly reduced the formation of Pol II/HIF1α complexes on the HIF1 target gene promoters (Fig. 9A). This could not be explained by USF2’s role in binding to HBS and activating HIF1 target genes under normoxia [43] as RCC4 cells exhibit constitutive HIF activity.

**Figure 10:**
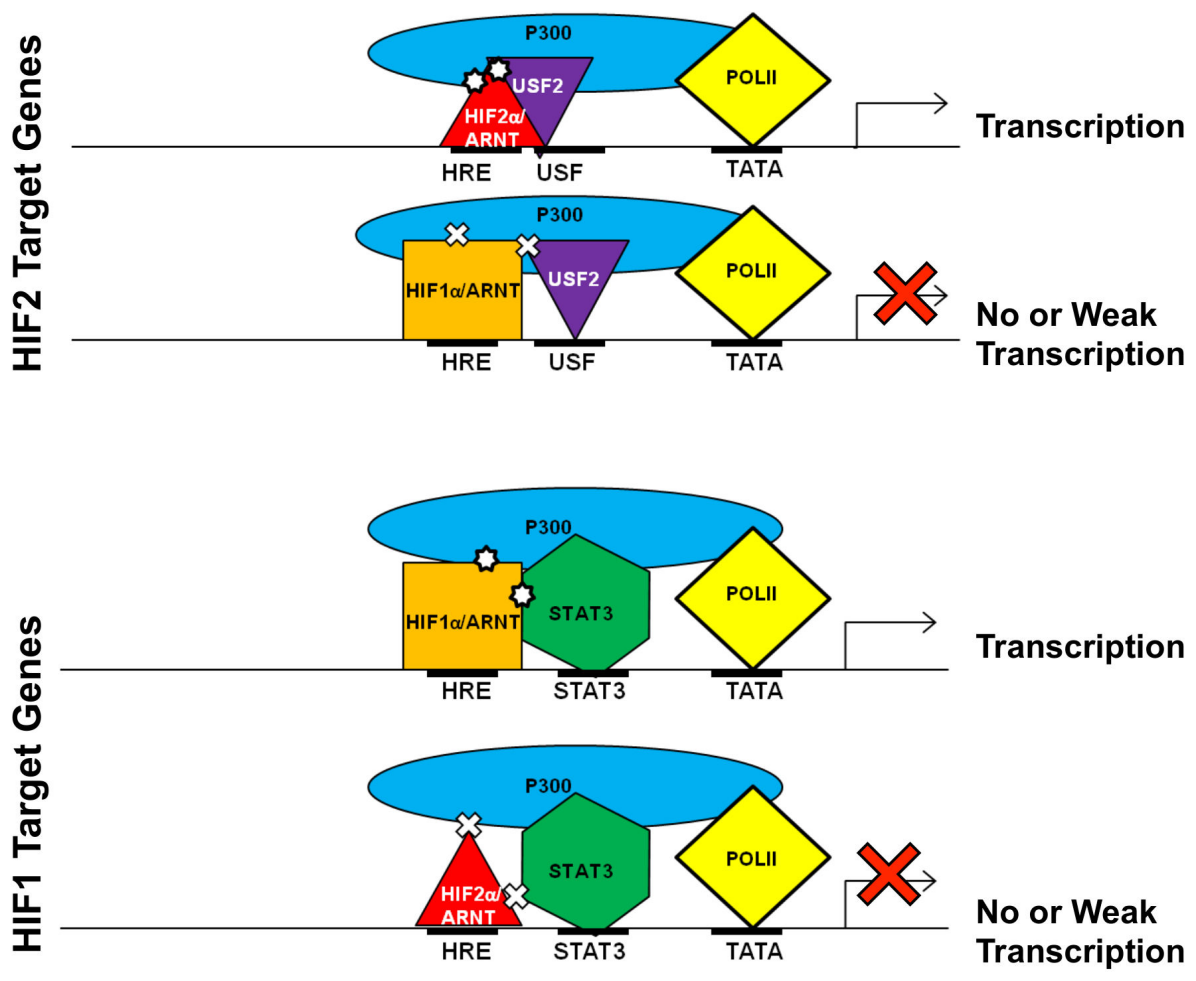
Model for HIF target gene specificity. Although HIF1α/ARNT and HIF2α/ARNT lack binding specificity, HIF1α/ARNT and HIF2α/ARNT activate unique target genes. Other transcription factors such as STAT3 and USF2 are required for HIF1 or HIF2 target gene activation, these HIF1 or HIF2 specific trascription partners contribute to HIF target gene specificity by mechanisms including: 1) A specific/preferential binding of STAT3 or USF2 to the promoters of HIF1 or HIF2 target genes. 2) Specific physical and functional interaction of HIF1α with STAT3, or HIF2α with USF2. and 3) HIF1α/ARNT specific transcription partners such as STAT3 or HIF2α/ARNT specific transcription partners such as USF2 are required for the formation of the functional transcription complexes on the promoters of HIF1 or HIF2 target genes respectively.

Thus, our results suggest that although HBSs for HIF1 or HIF2 target genes are identical, HIF1 or HIF2 target gene promoters contain binding sites for HIF1 or HIF2 specific transcription partners. The specific/preferential binding of the HIF1 or HIF2 transcription partners such as STAT3 or USF2 to the promoters of HIF1 or HIF2 target genes as well as the specific functional and physical interaction of HIF1α with HIF1 transcription partners including STAT3 or that of HIF2α with HIF2 transcription partners including USF2 are important for the formation of transcriptionally active Pol II-containing complexes specifically on the promoters of HIF1 or HIF2 target genes support the following model for HIF target gene specificity. For a HIF2 target gene, although both HIF1α and HIF2α can potentially bind to the HBS of HIF2 target gene promoters, only HIF2α can activate HIF2 target genes since HIF1α cannot interact with the HIF2α specific transcription partners such as USF2 to form a stable enhanceosome complex containing USF2, p300/CBP, Pol II and others (Figure 10). For a HIF1 target gene, although both HIF1α and HIF2α can potentially bind to HIF1 target gene promoters, only HIF1α can activate HIF1 target genes since HIF2α cannot interact with HIF1α specific transcription partners such as STAT3 to form a stable enhanceosome complex containing STAT3, p300/CBP, Pol II and others (Figure 10). We speculate that endogenously, the activating HIF may disassociate from the elongating Pol II complex after it is cleared from the promoter. Displacement of this functional complex from the HIF target gene promoter may result in unoccupied HBSs that may be quickly bound by an abundance of HIF protein under endogenous hypoxia. Thus, although both HIF1 and HIF2 can be detected on the HIF target gene promoter, only one HIF is productive in gene activation.

In summary, we present for the first time, insights into a molecular mechanism contributing to HIF target gene specificity. Additionally, identification of other transcription factors that are critical for HIF target gene induction and for HIF target gene specificity provides the foundation necessary to specifically inhibit HIF1 or HIF2 activity in treatment of solid tumors. Development of such HIF-specific therapies would undoubtedly prove beneficial for the optimal inhibition of tumorigenesis in a number of cancers as HIF2 function has been shown to be required for the development of hepatic and renal tumors in mouse models [44–46], but inhibit lung cancer growth [47], while HIF1 has been shown to inhibit growth of renal tumors [48], but to promote tumorigenesis of colon cancers [49]. Thus, our report not only increases our understanding of the molecular hypoxia response but also has practical implications for the rational design of anti-cancer therapies.

## Supporting Information

Figure S1 **The -499 and -464 STAT3 binding sites are functional in the CA9 promoter.** **A**) Schematic presentation of the CA9 promoters of -506/+25 and -171/+25 with potential STAT3, USF2 and HIF binding sites indicated. **B**) Fold of induction of CA9/Luc reporters activated by the indicated plasmids. CA9 -171/+25 was not activated by STAT3C and not cooperatively activated by HIF1αTM+STAT3C.
(TIFF)

Table S1 **The primers used in q-PCR to detect mRNA or genomic DNA in ChIP.** All these primers were tested for specificity and amplification efficiencies.
(TIFF)
