# Supplementary figures and images for: STAT3 or USF2 Contributes to HIF Target Gene Specificity
Source: PLoS One. 2013 Aug 21;8(8):e72358. doi: 10.1371/journal.pone.0072358 (PMC3749168; doi:10.1371/journal.pone.0072358)

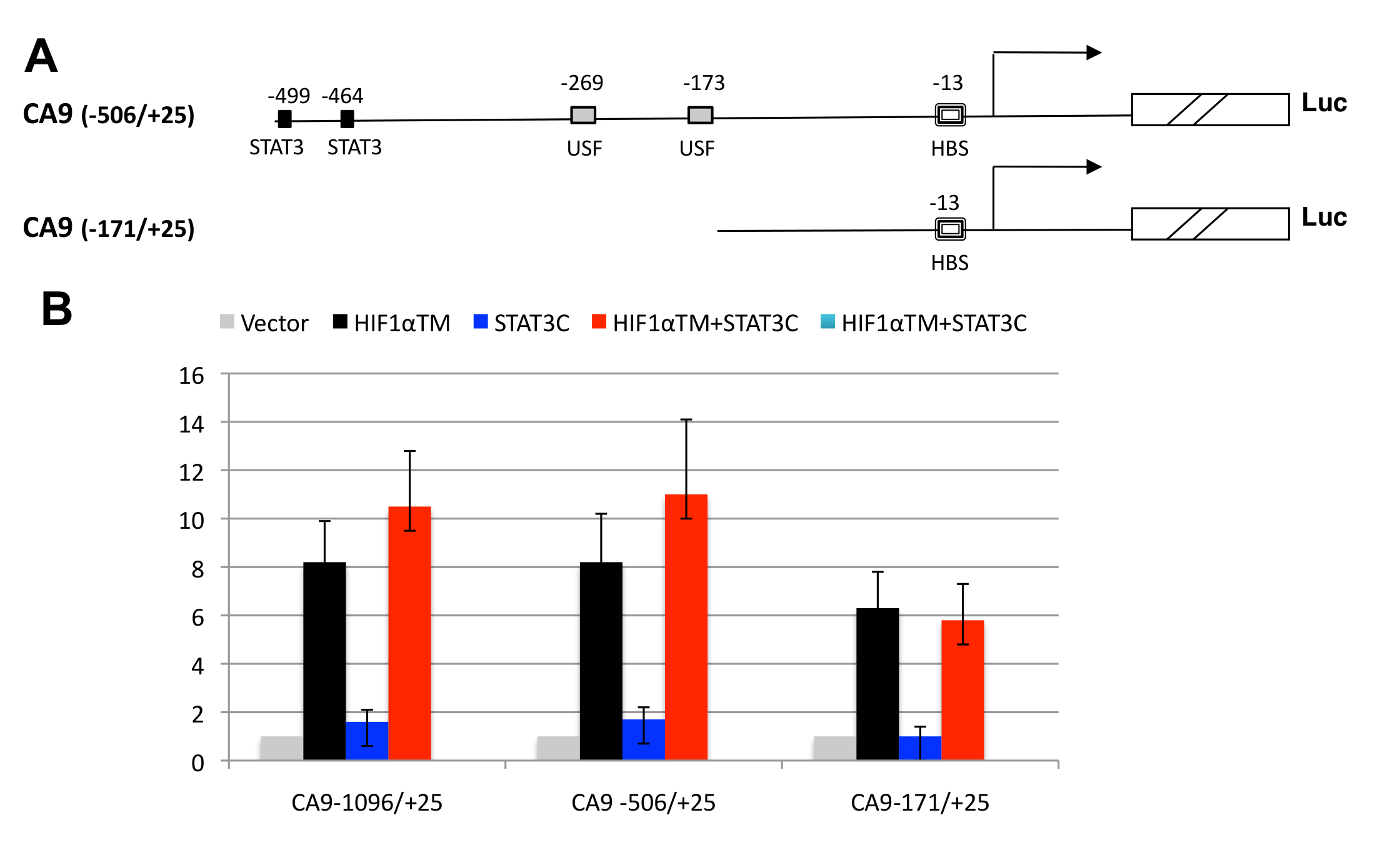

Supplement: Figure S1 — The -499 and -464 STAT3 binding sites are functional in the CA9 promoter. A) Schematic presentation of the CA9 promoters of -506/+25 and -171/+25 with potential STAT3, USF2 and HIF binding sites indicated. B) Fold of induction of CA9/Luc reporters activated by the indicated plasmids. CA9 -171/+25 was not activated by STAT3C and not cooperatively activated by HIF1αTM+STAT3C. (TIFF) [file pone.0072358.s001.tiff]

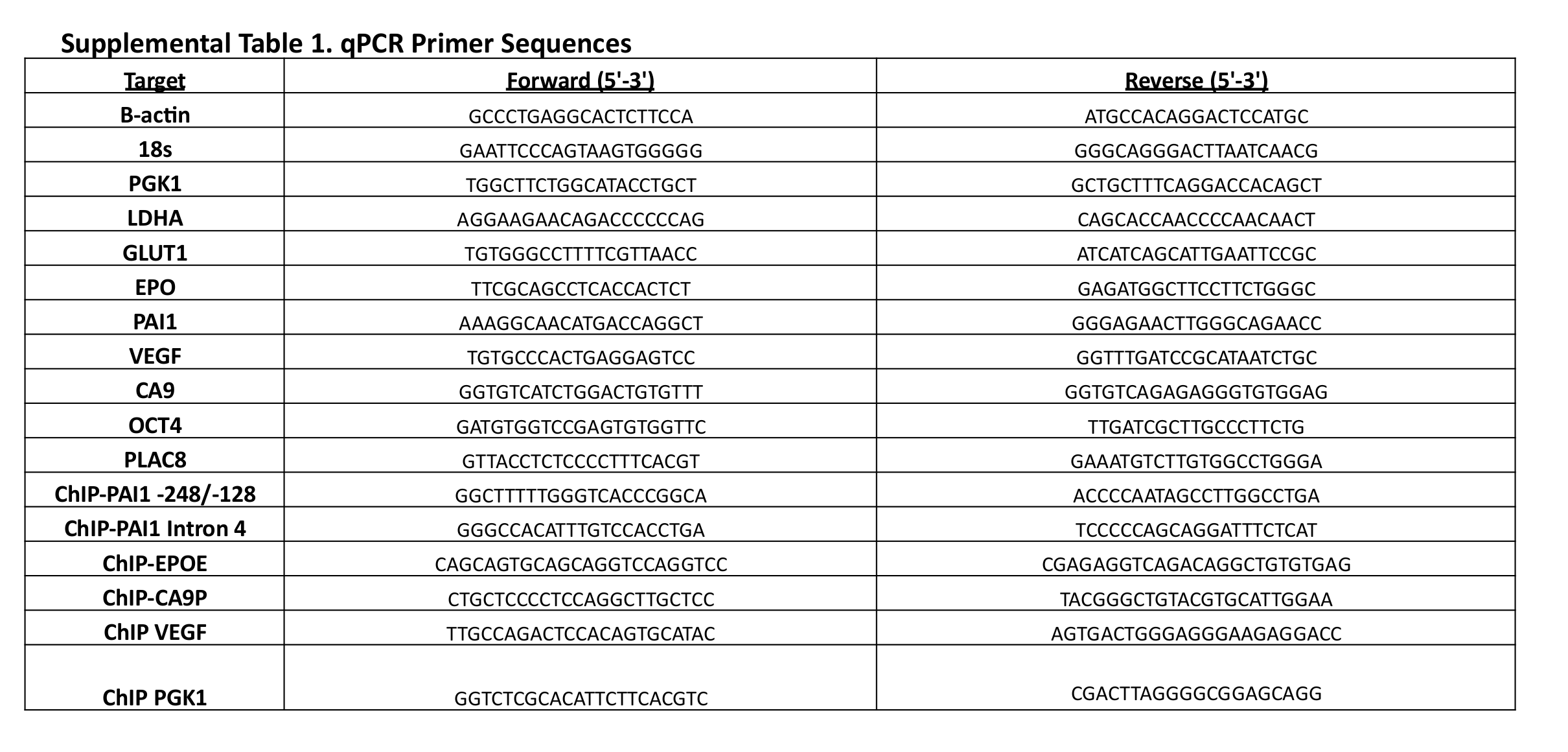

Supplement: Table S1 — The primers used in q-PCR to detect mRNA or genomic DNA in ChIP. All these primers were tested for specificity and amplification efficiencies. (TIFF) [file pone.0072358.s002.tiff]
